# A history of addiction through the six editions of Kandel’s *Principles of Neural Science* and their scientific context

**DOI:** 10.3389/fnbeh.2026.1755832

**Published:** 2026-04-08

**Authors:** Paula Santiago-Martinez, Oliver L. A. Hertog, Andrés Moncada-Parra, Ian Morales-Gutierrez, Diego Egaña-Yin, Javier Bernacer

**Affiliations:** 1 School of Medicine, University of Navarra, Pamplona, Spain; 2 Mind-Brain Group, Institute for Culture and Society (ICS), University of Navarra, Pamplona, Spain; 3 International Center for Neuroscience and Ethics (CINET), Tatiana Foundation, Madrid, Spain

**Keywords:** drug addiction, Eric Kandel, habit, history of neuroscience, relapse, substance abuse, tolerance

## Abstract

This review aims to explore the evolution of research on the neurobiology of addiction across the six editions of Eric Kandel’s *Principles of Neural Science*, one of the most comprehensive and well-known textbooks on neurobiology, published in 1981, 1985, 1991, 2000, 2012, and 2021. To encourage a critical reading of this historical review, we also summarize the state of the art in addiction research in the years preceding the publication of each edition. Even though addiction was mentioned as a crucial societal problem since the beginning, the manual did not explain it until the fourth edition. Decades before, several psychological hypotheses had already been integrated with neurobiology, emphasizing the role of dopamine and other biogenic amines, involvement of forebrain and mesencephalic areas, and pinpointing the neural correlates of psychological terms such as tolerance, dependence, craving, and others. Progressively, the neurobiological description of addiction transitioned from the hypothalamus to the basal ganglia, and, conceptually, from homeostasis to motivation, learning, and habit acquisition. Our intention with this review is to assess the evolution of addiction research in neuroscience, and also to show the strengths and weaknesses of how state-of-the-art research is integrated into specialized textbooks.

## 1 Introduction

The *Principles of Neural Science*, edited by Eric Kandel and collaborators, is the most widely recognized handbook of neurobiology. According to the publisher of the most recent three editions, McGraw-Hill, this property continues to sell the most units in print compared to all other neurology titles offered by the publisher, being one of the few manuals that still sells substantial print units. Its revenue is above one million dollars, and all editions have sold over 35,000 units per year. There are three editions initially sold by Elsevier (Kandel et al., 1991; Kandel and Schwartz, 1981, 1985), and the latter three editions were published by McGraw-Hill (Kandel et al., 2000, 2013, 2021). As stated in the Preface of the first edition, “*Principles of Neural Science* is designed as an introductory text for students of biology, behavior, and medicine. Our overall goal is to convey the interest and excitement surrounding the recent attempts to apply cell-biological techniques to the study of the nervous system, its development, and its control of behavior” (p. xxix). Thus, this is one of the most comprehensive and respected handbooks for learning the neural bases of behavior, including mental disorders.

Our historical review aims to examine the evolution of the concept of addiction over the last half-century through the six editions of Kandel’s *Principles*. To promote a critical view, we have also analyzed the state of the art in addiction research before each edition’s publication, allowing the manual’s contents to be contrasted with the scientific knowledge of that time. We decided to concentrate on addiction for several reasons. First, the Preface of the 1st edition of Kandel’s manual presents it as a societal problem beyond medicine. Today, we all recognize addiction as a public health problem involving psychiatry, psychology, neuroscience, and other fields of knowledge. We believe that a critical analysis of the transition between both conceptual positions is an interesting approach to better understand addiction research, past and present, and to identify future challenges. Second, substance use and abuse, including alcohol, account for over 3.2 million deaths around the globe, while 296 million people aged between 15 and 64 years use psychoactive drugs (World Health Organization, 2024). According to the United Nations, illegal drugs are the source of immense human suffering, mainly affecting young people, and their use increases every year. Therefore, it is a worldwide public health issue that is growing each year. Finally, addiction has experienced a change in the last decade: classical behavioral addictions like gambling have met new ones, namely gaming, problematic Internet use, social media addiction, compulsive buying, or pornography use. These are more problematic, since younger people (including children) can have an easy access to the products, and are especially vulnerable partly because of an ongoing brain maturation. Therefore, a conceptual review of the neurobiology of addiction in recent history could help researchers predict future challenges in this field.

In this review, we searched the detailed index and glossary of each edition of *The Principles of Neural Science* (including also the *Cellular basis of behavior*, authored by Kandel and published in 1976, which can be considered as a prequel to the *Principles*) (Kandel, 1976) to look for mentions of addiction or related concepts. Additionally, we analyzed the Prefaces of all editions to identify any references to the topic. Once the chapter or subsections were selected, we read them in depth and extracted the main ideas. The state of the art for each edition was selected by quasi-systematically searching PubMed for relevant articles on the neurobiology of addiction. The intended search included the terms “neurobiology of addiction” (between quotes), with the article type limited to Consensus Development Conference, Consensus Development Conference NIH, Editorial, Guideline, Practice Guideline, Review, Scoping Review, Systematic Review. However, since the term “neurobiology” was hardly used at the time of the first editions, we used more flexible terms such as “brain” and “addiction” (for the 1st, 2nd and 3rd editions), “neurobiology” and “addiction” (for the 3rd and 4th editions), and “neurobiology of addiction” (for the 5th and 6th editions). In all cases, we also included some relevant review articles found in the bibliographies of the selected publications. Year of publication was limited to 1977–1980 (state of the art before the 1st edition), 1981–1984 (2nd edition), 1987–1990 (3rd edition), 1996–1999 (4th edition), 2008–2011 (5th edition), 2017–2020 (6th edition). In any case, our goal was not to conduct a systematic review, but to provide a broad (though unbiased) outline of research on addiction for each time period. Consequently, not all output articles were analyzed in depth, and some others that were historically relevant and contained new hypotheses were included, even though they were published in the interim years (for example, Robinson and Berridge, 1993, introducing the Incentive Salience Theory, or Everitt and Robbins, 2005, presenting the habits-compulsions theory).

Overall, this historical review aims to illustrate the evolution of neurobiology in addiction over the last four decades and its representation in one of the most widely consulted textbooks by junior and senior behavioral neuroscientists. From this critical view of the past, we may learn some lessons for the present and future of addiction research and how it is systematically presented to students and scientists.

## 2 The concept of addiction in Kandel’s *Principles of Neural Science*

The *Principles of Neural Science* is a manual aimed at students and scholars interested in neurobiology. Therefore, it is expected to find a “reductionist” approach biased toward basic biology instead of psychology, ethology, etc. There are several ways to understand the relationship between brain and behavior, especially in the case of addiction, where purely neural explanations are contrasted with more ecological views. A description of the mind-body problem and related topics is beyond the scope of this article. However, it is essential to state the position of the *Principles* on this topic clearly.

Before the *Principles of Neural Science*, Kandel published the *Cellular Basis of Behavior* (Kandel, 1976), which can be considered a prequel to his famous manual. It is intended to be “usable by undergraduate and graduate students (…) as well as to provide an overview of the neurobiology of behavior for scientists in other fields” (p. xv). Firmly grounded in evolution, the history of modern psychology, and invertebrate neurobiology, it is a comprehensive compendium of the neural science of its time and its relationship to behavior. How does Kandel understand the connection between neurobiology and behavior? The opening sentence of the initial chapter in the first edition of the *Principles* is eloquent about the perspective that the author of this collective work takes on behavior: “The key philosophical theme of modern neural science is that all behavior is a reflection of brain function (…) the mind represents a range of functions produced by the brain” (Kandel and Schwartz, 1981, p. 3). The third edition is more succinct about this: “The goal of neural science is to understand the mind” (Kandel et al., 1991, p. xxxix). Also, he advocates for a strong localizationism of cognitive and affective functions, as well as character traits: “Why has the evidence for localization, which seems so obvious and compelling in retrospect, been repeatedly rejected in the past?” (Kandel et al., 1991, p. 11). The optimistic attitude toward neuroscience is remarkable, even though neuroimaging –the possibility of studying the human brain in live participants and in relation to behavior– was still absent: “The excitement in neural science today resides in the conviction that the tools are at last in hand to explore the organ of the mind, and with that excitement comes the optimism that the biological basis of mental function will prove to be fully understandable” (Kandel et al., 1991, pp. 11–12). Therefore, the *Principles* must be read as a manual of neurobiology whose intention is to explain the mind and behavior, based on neural function, as accurately as possible.

### 2.1 A field to be discovered in the prequel and the first three editions (1976–1991)

Going back to the *Cellular Basis of Behavior* (published in 1976), addiction and related terms –alcoholism, substance use, dependence, etc.,– are not mentioned throughout the text, even though learning is considered a key topic of behavior. The book includes a complete description of the experiments done by authors such as Pavlov, Thorndike, Lashley, Tolman, and Thorpe. Conditioned responses, habituation, sensitization, reinforcement, and similar terms are described in detail, but are unrelated to addiction. The connection between this condition and aberrant learning had not yet been proposed. The last chapter is dedicated to the “Implications for the Study of Abnormal Behavior.” Citing Claude Bernard, Kandel states that “disease states are often extreme manifestations of normal processes and follow lawful patterns that can be successfully analyzed with biological techniques (…) As a result, normal cellular functioning and its alteration in disease have come to be viewed within a common, biological framework” (Kandel, 1976, p. 653). However, he is cautious about the application of this perspective to human behavior: “This framework, which encompasses both normal and abnormal behavior, has not yet been firmly established within psychiatry” (p. 653). Kandel draws the classic distinction between neuroses and psychoses, which involve one or more of the following “psychological states” with a varied degree of severity: anxiety, depression, mania, paranoia, delusion, hallucination, and thought disorders. When describing the disturbances of motivational states and stress, which disrupts homeostasis, many descriptions evoke addictive behaviors, albeit they are not mentioned: “For example, a strong stimulus that usually produces a large response may fail to do so; a constant stimulus that normally produces and unvarying response may produce variations of that response; or a change in the stimulus may not be reflected by a change in the response” (Kandel, 1976, p. 658). Following a neurobiological description of these disturbances in *Aplysia*, Kandel relates them to obesity and anorexia in humans, but not to addiction.

Following this line, the first three editions of the *Principles* do not deal explicitly with addiction. However, there is a fascinating mention in the first edition’s preface (published in 1981): “Not needed only for clinical application, neural science is required for understanding human behavior, because all behavior is an expression of neural activity. Beyond medicine, in society at large, the problems of crowding, addiction, violence, and war revolve around the nature of human beings. Any intelligent solutions to the enormous problems of human behavior, individual and collective, must benefit from greater knowledge of neural function. Many of these problems are not now in the immediate domain of neural science, but progress is rapid and we can hope that neural scientists will soon be able to contribute directly to understanding them” (Kandel and Schwartz, 1981, p. xxxi). Addiction is viewed as one of the main societal problems beyond medicine at the time, with the firm conviction that it would be scientific soon. This sentence remains unchanged in the second edition, but in the third, these topics are replaced by a focus on the mind and consciousness as the “frontiers of biology” (Kandel et al., 1991, p. xl).

Returning to the first edition, mental illness is discussed in the context of schizophrenia (as the primary example of thought disorder) and depression (as the paradigm of affective disorder). Regarding topics potentially related to addiction, there are extensive descriptions of associative and non-associative learning, habituation, and a Pavlovian description of memory. Chapters 37 and 38, signed by Irving Kupfermann (Kandel’s long-standing collaborator, biologist and expert in *Aplysia*), describe the limbic system and motivation. The author explains the role of these systems in maintaining homeostasis, highlighting hormonal responses and their relationship to pain, pleasure, learning, and emotional behavior in general. Nowadays, we naturally relate these topics to addiction, and the link was already proposed in the scientific literature, as we will see in the next Section. Interestingly, the earliest edition of the *Principles* describes behavior reinforcement through intracranial self-stimulation of the hypothalamus, discovered by Olds and Milner in 1954 (Olds and Milner, 1954). In Kandel’s *Principles*, this idea is connected to normal behavior: “Support for this idea has come from the subsequent observations that many of the points in the brain that are effective in producing reward also stimulate complex behavioral patterns such as feeding and drinking” (Kandel and Schwartz, 1981, p. 459).

The second edition (also written by Irving Kupfermann, published in 1985) shows similar ideas about motivation, learning, reinforcement, and intracranial stimulation. However, some additions in these chapters tighten the gap between motivation and addiction, even though this disorder is still unmentioned. Motivational states are defined as the “internal conditions that arouse and direct voluntary behavior” (Kandel and Schwartz, 1985, p. 626). Some are specifically called “drives” (i.e., urges or impulses based upon bodily needs). However, not all drives address physiological needs, such as curiosity. In this case, no homeostasis imbalance should be corrected. Sometimes, motivational states are just the interaction between external and internal stimuli. Further, hedonic factors (that is, pleasure) can regulate motivated behaviors. This is where the topic is approaching addiction, although the explicit connection is not yet made in the text. According to this edition, the neural bases of pleasure are poorly understood. Still, it is hypothesized that these mechanisms overlap or even coincide with brain processes concerned with reward and reinforcement of learned behavior. Biogenic amines, and more precisely dopamine, are already suggested as candidate neurotransmitters.

In the third edition (published in 1991), addiction is also not mentioned. The chapter on motivation (also by Irving Kupfermann) explains, as in the previous editions, that motivational states can be regulated by factors other than tissue needs. In this case, the author mentions ecological constraints, anticipatory mechanisms, and, like in the previous edition, hedonic factors. The first aspect involves cost-benefit functions that maximize food intake (or any similar feature) in response to the animal’s external conditions. The second relates to circadian rhythms, which enable animals to anticipate their basic needs. Hedonic factors are explained in the same words as in the previous edition. However, there is an important addition in the final section regarding intracranial stimulation. After explaining the basics as in the second edition, the author adds: “Stimulation of the nucleus accumbens is also reinforcing. In fact, addictive drugs such as cocaine may induce euphoria by enhancing the action of dopamine at the nucleus accumbens, which receives substantial dopaminergic input” (Kandel et al., 1991, p. 759). Therefore, even though it is still absent in the book, addiction is progressively emerging as a topic of interest in the chapter on motivational states.

In conclusion, addiction is a nearly unexplored topic in the three initial editions of Kandel’s *Principles of Neural Science* (Kandel and Schwartz, 1981, 1985; Kandel et al., 1991). Its scarce mentions are linked to motivation and homeostasis. Dopamine is proposed as a candidate neurotransmitter to be involved in the overall process of motivation, and the nucleus accumbens is explicitly mentioned as the target of drugs of abuse, even though its role in addiction is unmentioned. From 1991 to 2000, scientific advances in neuroscience and psychology consolidated the presence of addiction in the book. Finally, it was honored with its own chapter (*ex aequo* with “motivation”) in the fourth edition.

### 2.2 The progressive consolidation of addiction in the fourth, fifth, and sixth editions (2000–2021)

Nine years after the publication of the third, the fourth edition came out (in 2000, the same year that Eric Kandel received the Nobel Prize), including a chapter about “Motivational and Addictive States” (chapter 51), authored by Irving Kupfermann, Eric Kandel, and Susan Iversen (neuropsychopharmacologist). For the first time, addiction was considered a topic in this manual, tightly linked to motivation, which has been seen as the engine that initiates, sustains, and directs behavior toward specific goals. Traditionally, the study of motivation was narrowly focused on the drive to satisfy physiological needs, such as hunger, thirst, and the avoidance of pain. These needs were conceptualized within a framework of discomfort and relief, in which the organism’s behavior was seen as a response to restore physiological balance, namely homeostasis. Early theories inferred drive states solely from observable behaviors. However, advancements in neuroscience shifted this perspective to include the role of control systems, conceptualizing drive states as complex homeostatic reflexes that integrate sensory, cognitive, and emotional information.

Even though the fourth edition did not have a systematic analysis of addiction, it started to show the relationship between drugs of abuse, reinforcement, and the limbic system. Craving, dependence, and tolerance were also briefly discussed. Neuroimaging (PET) studies began to link motivation to brain regions responsible for various forms of memory, including working, episodic, and emotional memory (see Box 51–1 in the 4th edition, Kandel et al., 2000). Studies demonstrated that activity in these regions was directly linked to the intensity of craving, particularly in the context of substance use disorders. This evidence suggested that the mechanisms underlying memory processing were intricately tied to the experience of craving, highlighting a potential overlap between the neural circuits involved in memory and those mediating the effects of addictive substances. Specifically, brain regions such as the hypothalamus, which play a critical role in reward processing and reinforcement learning, appeared to intersect with these mechanisms, reinforcing learned behaviors associated with drug use.

When examining disorders of these neural circuits, addiction stood out as a primary example of how the reward pathway can be pathologically altered. Also, motivation, reinforcement, and addiction were linked for the first time (in this manual) with dopaminergic pathways. Different drugs of abuse cause similar addictive states by acting on the brain circuits that control reward and motivation, namely mesolimbic dopaminergic pathways, acting on transporters and receptors. Addiction to opiates, specifically, is mentioned in chapter 24, devoted to the perception of pain. Going back to chapter 51, it concludes with the concepts of tolerance and dependence. “Tolerance refers to progressive adaptation to the dosage that produces euphoria” (Kandel et al., 2000, p. 1011). It develops as the brain adapts to the repeated presence of a drug, diminishing its effects over time and necessitating higher doses to achieve the same level of reward. Dependence, on the other hand, “refers to the negative visceral consequences of withdrawal of the drug, such as nausea” (ibidem). It reflects the physiological and psychological reliance on the drug, characterized by withdrawal symptoms when its use is reduced or stopped. Together, these features create a cycle of compulsive drug-seeking and use that defines addiction. The interplay of reinforcement, tolerance, and dependence underscores the complexity of addiction, needing a multifaceted approach to its study and treatment.

In the fifth edition (published in 2012), chapter 49 was devoted to “Homeostasis, motivation and addictive states,” authored by Peter B. Shizgal (behavioral neurobiologist) and Steven Hyman (molecular psychiatrist). Addiction was becoming a well-established and independent concept in neural sciences. To our knowledge, this edition is the first to include an explicit definition of addiction: “compulsive drug use despite significantly negative consequences” (Kandel et al., 2013, p. 1105). Essentially, drugs become the only life goal and forego necessities, even if they affect the quality of life. However, the main complication of drug use is persistence. Not only is quitting complicated, but there is a prevalent (and even permanent) risk of relapse by exposure or cues after usage has ended. The authors pinpointed a concept that was just touched upon in previous editions: reward, which is defined as objects, stimuli, or activities that have a net positive effect.

The chapter subsequently explains in detail the autonomic systems that regulate feeding and drinking, which introduces motivational states. These influence goal-directed behavior through internal and external stimuli, and may serve behaviors beyond homeostasis, such as sexual arousal. These stimuli, whether internal or external, homeostatic or non-regulatory, serve as rewards, guiding the animal’s goal selection. Therefore, the reward system is fundamental for goal-directed behavior. At a neurobiological level, the strongest responses to the reward system come from the medial forebrain bundle and longitudinal fiber bundles in the midline of the brain stem. They are enhanced by an increase in transmission from cholinergic cells in the laterodorsal tegmental and pedunculopontine nuclei of the hindbrain. As a result, studies have shown that stimulation of specific brain regions makes animals willing to forgo necessities to continue the stimulation.

Remarkably, in conflict with some psychological theories of addiction (see Everitt and Robbins’ below), the authors emphasize that drug abuse and addiction are goal-directed behaviors. They explain how drugs of abuse affect the dopaminergic pathway by increasing extracellular dopamine in the nucleus accumbens: “Thus, psychotropic drugs that do not produce significant dopamine release in the nucleus accumbens are not addictive” (Kandel et al., 2013, p. 1106). However, they clarify that opiates follow dopamine-independent mechanisms. Wolfram Schultz’s experiments about the role of dopamine in reward consumption and anticipation are briefly mentioned (Schultz, 1997). Finally, this edition highlights the long-term neural effects of addictive drugs. While the previous edition introduced craving, tolerance, and dependence, this one also includes sensitization: “When effects grow stronger with repeated drug use, they are said to undergo sensitization. For example, the locomotor activity produced by amphetamine or cocaine increases with repeated use of the drug” (Kandel et al., 2013, p. 1109).

In the sixth edition (published in 2021), chapter 43 is entitled “Motivation, Reward and Addictive States,” and authored by Eric J. Nestler (molecular psychiatrist) and C. Daniel Salzman (cognitive neuroscientist). Note that “homeostasis” (now explained in chapter 41) is substituted for “reward” in the title. Also, addiction is briefly described in the chapter about the basal ganglia (chapter 38), authored by Peter Redgrave (neurobiologist) and Rui M. Costa (computational neuroscientist). After presenting the role of these structures in action selection and reinforcement learning, chapter 38 discusses whether disorders of the basal ganglia are disorders of selection. Among them, addictions are presented as disorders of reinforcement mechanisms and habitual goals. In this chapter, addiction is defined as a dramatic dysregulation of motivational selections, “caused by an exaggerated salience of addiction-related stimuli, binge indulgence, and withdrawal anxiety” (Kandel et al., 2021, p. 950). It involves changes in dopaminergic and opioid transmission. Since these neurotransmitter systems are related to reinforcement, addictive cues show an enhanced salience to capture behavior. In contrast with the previous edition, the authors clarify that drug-seeking may involve complex goal-directed behavior, although overall drug acquisition may be a stimulus-driven habit.

Chapter 41, like in previous editions, starts explaining the role of internal and external incentive stimuli in motivational states. Regarding rewards meeting homeostatic and non-regulatory needs, the main novelty in this edition is the authors’ claim that certain rewards and goals extend into longer timescales. Some motivational states entail more complex long-term goals driven by incentive stimuli, non-regulatory needs, such as finding a romantic partner or achieving a professional goal. In these scenarios, actions are not immediately rewarded, and the motivational states must be sustained across challenging circumstances to achieve certain goals. Regarding neuroanatomy, the reward system provides a biological substrate for goal selection, which involves assessing risks, costs, and benefits. Thus, there are neural mechanisms responsible to weigh the costs and benefits of behavior leading to a goal. Pathologies such as addiction hijack these reward systems, resulting in maladaptive behavior. Still, the work by Olds and Milner (1954) about rewarding self-intracranial stimulation is presented as a key experiment in this respect. Before discussing addiction, the authors explain more extensively Wolfram Schultz’s experiments about the role of dopaminergic neurons in learning (Schultz, 1997).

Drug addiction is defined as “a chronic and sometimes fatal syndrome characterized by compulsive drug seeking and consumption despite serious negative consequences such as medical illness and inability to function in the family, workplace, or society” (Kandel et al., 2021, p. 1069–1070). The authors highlight that only a few chemicals are drugs of abuse and, being diverse in their structure, they all target the reward system, which involves not only dopamine, but also glutamate and GABA. A new fact stated in this edition is that about 50% of the risk for substance addiction is genetic. The concepts of tolerance, sensitization, dependence, and withdrawal are also defined in the sixth edition. After this, the authors explain in depth some molecular processes that may explain addiction, such as upregulation of the cAMP-CREB pathway (already mentioned in the previous edition), and induction of ΔFosB transcription factor (new here). Then, and also for the first time, synaptic (long-term potentiation and depression) and whole-cell plasticity in the ventral tegmental area and nucleus accumbens are explained in the context of addiction. Circuit plasticity involving the prefrontal cortex, hippocampus, amygdala, thalamus, nucleus accumbens, and midbrain is briefly mentioned as an emergent field in addiction, as well as the impact of drugs on glia and endothelial cells.

Finally, as an important landmark, the last section introduces “natural addictions.” The brain’s reward system evolved to encourage the pursuit of natural rewards, such as food, reproduction, and social interaction. However, some individuals exhibit compulsive engagement in these otherwise normal activities, stepping into overeating, uncontrollable shopping, gambling, video gaming, or sexual behavior, in ways that strongly resemble drug addiction. Researchers are exploring whether these “natural addictions” (also known as behavioral addictions) are driven by similar molecular, cellular, and circuit-level changes as those seen in substance abuse. One possibility is that certain people, due to genetic or non-genetic vulnerabilities, experience excessive activation of reward pathways, repeatedly seeking the initial pleasure even in the face of negative consequences. Studying natural addictions is more complex than studying drug use, partly because animal models are not straightforward to develop. With that said, human brain imaging studies increasingly suggest that both drug addictions and behavioral addictions involve similar dysregulation of the brain’s reward circuitry.

In conclusion, the neurobiological study of addiction has become more established through the successive editions of Kandel’s *Principles of Neural Science*. Its relationship with reward, reinforcement learning, action selection, and dopamine has been increasingly grounded. Furthermore, the level of detail is higher, involving genetics and describing precise molecular pathways that affect learning. Remarkably, the field of behavioral addictions is starting to emerge; these are probably the most prevalent in present times.

After summarizing the concept and study of addiction through the six editions of Kandel’s manual (see Table 1 for a summary of the main ideas included in each edition), we will present the scientific context around this topic in the years before the publication of each edition. Thus, we outline key scientific publications that reflect the state of the art in addiction research for each time period. By doing so, we aim to provide a contextual reading of the manual, emphasizing its contribution to this field of knowledge.

**TABLE 1:** Summary of the key ideas contained in each edition of Kandel’s manual, including the *Cellular basis of behavior.*

Edition	Summary of contents related to addiction
Cellular Basis of Behavior (1976)	Addiction and related terms are absent. Focus on learning without link to addiction. Last chapter explores abnormal behavior, but addictive phenomena are not discussed.
Principles of Neural Science, 1st ed. (1981)	Addiction is not analyzed but explicitly mentioned in the preface as one of society’s major problems. The book discusses motivation, emotion, pain, and pleasure in the context of homeostasis and reinforcement, but not of addiction. Intracranial self-stimulation is described as a form of reward, but not linked to addiction.
2nd ed. (1985)	Addiction remains unmentioned. Motivation is defined as internal states that arouse and direct behavior. Pleasure is introduced as a regulator of motivated behavior. Dopamine and other biogenic amines are proposed as potential mediators of reward and reinforcement.
3rd ed. (1991)	Still no explicit mention of addiction. The discussion of motivation is expanded to include ecological and anticipatory regulation and hedonic influences. First mention of the nucleus accumbens and dopamine in the context of reinforcement. Addiction thus emerges implicitly, although not yet thematically developed.
4th ed. (2000)	For the first time, a chapter on “Motivational and Addictive States” is included. Addiction is explicitly introduced as a pathological alteration of reward and motivation circuits, particularly the mesolimbic dopaminergic system. Concepts such as craving, tolerance, and dependence are defined, highlighting overlap between reinforcement, learning, and addiction.
5th ed. (2012)	Addiction becomes an established topic. The chapter “Homeostasis, Motivation and Addictive States” details the neurobiology of reward, motivation, and relapse. Dopamine release in the nucleus accumbens is identified as essential for addiction, though opiates are noted to act via dopamine-independent mechanisms. Tolerance, dependence, and sensitization are described as key phenomena.
6th ed. (2021)	Addiction is deeply integrated into the neuroscience of motivation and reward, now separated from homeostasis in the title “Motivation, Reward and Addictive States.” Addiction is defined as a chronic syndrome of compulsive drug seeking despite severe negative outcomes. Molecular mechanisms are explained in detail. Synaptic and circuit plasticity are linked to addiction. Behavioral (“natural”) addictions are introduced as a new conceptual domain.

## 3 The scientific context of addiction for each edition of Kandel’s manual

### 3.1 State-of-the-art in the first edition (1977–1980)

**FIGURE 1:**
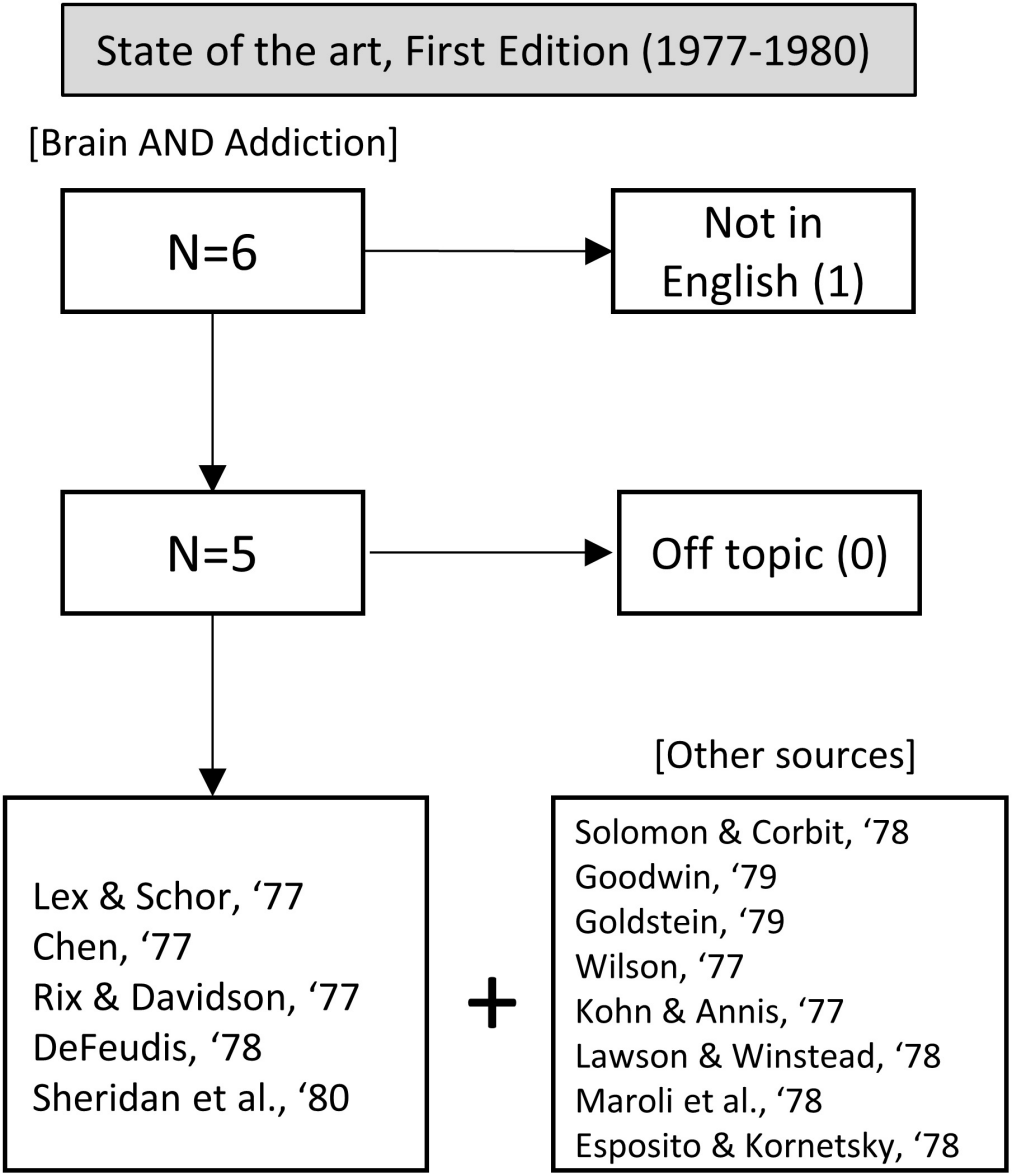
Flow chart showing article selection for the state of the art before the publication of the first edition of Kandel’s handbook.

As we mentioned in the Introduction, “neurobiology of addiction” was an inexistent field in those years. Therefore, our search included the terms “brain” and “addiction,” resulting in 6 articles. One of them was excluded because it was not in English. We complemented this search with other terms (such as “alcoholism”) to retrieve other potentially interesting sources. In total, we analyzed 13 articles (see Figure 1 for a flow chart of this process).

Regarding conceptual or theoretical frameworks, addiction was already perceived as a multifactorial issue, a “more general phenomenon” which goes beyond mere chemical imbalances and physiological causes (Solomon and Corbit, 1978, p. 12). As Goodwin put it, “Are the causes biological, sociological, or psychological? The fashion today is to say all three” (Goodwin, 1979, p. 161). As such, there was plenty of disagreement within the literature as to which component holds the most weight in the definition of addiction, whether it is a physical condition, a behavioral problem, or a question of upbringing or social environment. Following this line, Lex and Schor (1977) propose preliminary conjectures about possible neurobiological relationships among seemingly disparate phenomena: religious rituals, native curing therapies, and the pharmacodynamic, psychological, and sociocultural components of opiate addiction.

In this climate, several new theories were being proposed. For example, the first Opponent Process Theory “of motivation” was proposed by Solomon and Corbit, which explains addiction as a sequence of paired emotional and behavioral responses attributed to underlying physiological processes (which were still only vaguely identified). Behaviorally speaking, addiction is defined as a short hedonic, appetitive, State A (the initial “rush” of euphoria, which is positively reinforcing), followed by an opposite State B, which is slowly decaying, dysphoric and aversive, and ultimately a summation of both effects as the patient returns to their initial baseline neutral state. After repeated drug use, the B process strengthens, leading the patient to use the drug to elicit State A to escape State B; otherwise, increased drug use is attributed to negative reinforcement rather than positive reinforcement or pleasure-seeking (Solomon and Corbit, 1978). There was also a resurgence of older theories, such as the Homeostasis Hypothesis, originally proposed by Himmelsbach (1943), which suggested that the body adapts to drug use to maintain homeostasis. More physiologically focused theories questioned the concepts of dependence, tolerance, and withdrawal, challenging the view that they are key pillars of addiction. For instance, Goldstein (1979) used molecular techniques to examine the lipid composition of cellular membranes to operationalize tolerance and dependence as a function of membrane fluidity; he concluded that “functional tolerance” and “physical dependence” were two separate processes dissociated from addiction, as the characteristic behaviors of addiction can occur in their absence.

Regarding the neurobiology of addiction, it was focused on the role of specific neurotransmitters, primarily catecholamines, and their association with drug-taking behavior. Studies such as Maroli et al. (1978) and Esposito and Kornetsky (1978) relate catecholaminergic activity in the medial forebrain bundle to reinforcement of prolonged drug use, primarily dopamine and norepinephrine. Sheridan et al. (1980) propose that acetaldehyde, the main metabolite of ethanol metabolism, is neuroactive and will stimulate dopaminergic circuitry such as the mesolimbic reward pathway and the VTA, creating motivational and reward effects. It is suggested that acetaldehyde hydrate could act as a strong inhibitor of aldehyde dehydrogenase, slowing its breakdown and thereby increasing acetaldehyde levels, perpetuating these reinforcing effects. On the other hand, Rix and Davidson (1977) question whether GABAergic systems have a causal role in the genesis or maintenance of alcoholism or other drug-dependent states. Finally, Chen (1977) suggests the role of acupuncture to mitigate opiate withdrawal symptoms through enkephalin enhancement.

On the other hand, researchers focus rather on the etiology of addiction and on an ongoing “nature vs. nurture” debate. In other words, they discuss whether there is a biological and/or psychological vulnerability, or if addiction is an acquired condition. Goodwin, for example, through twin and adoption studies, suggests that alcoholism is primarily a genetically inherited condition whose “strongest predictor” is a family history of alcoholism (Goodwin, 1979). Wilson’s study sides rather with the “nurture” aspect of the debate, arguing that the “screwed-up” and “precipitated” alcoholics were determined by their environment (Wilson, 1977). DeFeudis gives a more neurobiological approach to the topic by focusing on environmental vulnerability factors in animal models. He suggests that “environmental impoverishment” (social isolation) may promote addiction by affecting neural pathways and drug metabolism (DeFeudis, 1978), such as by increasing sensitivity to morphine-induced analgesia. Other theories focus on the “nature” side of addiction, and the intrinsic personality-based vulnerability factors people may carry, which could predispose the initial use of a drug. Kohn and Annis (1977) use a scale of 4 constructs to determine a person’s tendency toward novelty seeking as a predictor of initial drug use. Additionally, Lawson and Winstead (1978) explain drug use as “temporary symptomatic relief” of the discomfort created by one’s internal stress (personality traits) and external stress (environment).

In sum, during these years, addiction is viewed as a multifactorial condition, with neurobiological correlates focused on catecholamines and endogenous opioids, such as enkephalin. The dominant theories were the opponent process and homeostasis. Several studies highlighted the importance of predisposing factors to addiction, both at genetic and personality levels, and others highlighted the role of education and social factors.

See Table 2 for a summary of the key findings on the neurobiology of addiction in this period.

**TABLE 2:** Key issues of the state of the art in the neurobiology of addiction before the publication of the first edition of Kandel’s handbook.

Issue	Main ideas	References
Frameworks and concepts	Multifactor phenomenon	Goodwin, 1979; Lex and Schor, 1977
	Opponent process	Solomon and Corbit, 1978
	Homeostasis	Goldstein, 1979
Specific NT or biological systems	Catecholamines	Maroli et al., 1978; Esposito and Kornetsky, 1978; Sheridan et al., 1980
	GABA	Rix and Davidson, 1977
	Enkephalin	Chen, 1977
Specific drugs	Alcohol	Sheridan et al., 1980; Rix and Davidson, 1977
	Opioids	Chen, 1977
Vulnerability factors	Genetics (family history)	Goodwin, 1979
	Environment	Wilson, 1977; DeFeudis, 1978
	Personality traits	Kohn and Annis, 1977; Lawson and Winstead, 1978

### 3.2 State-of-the-art in the second edition (1981–1984)

**FIGURE 2:**
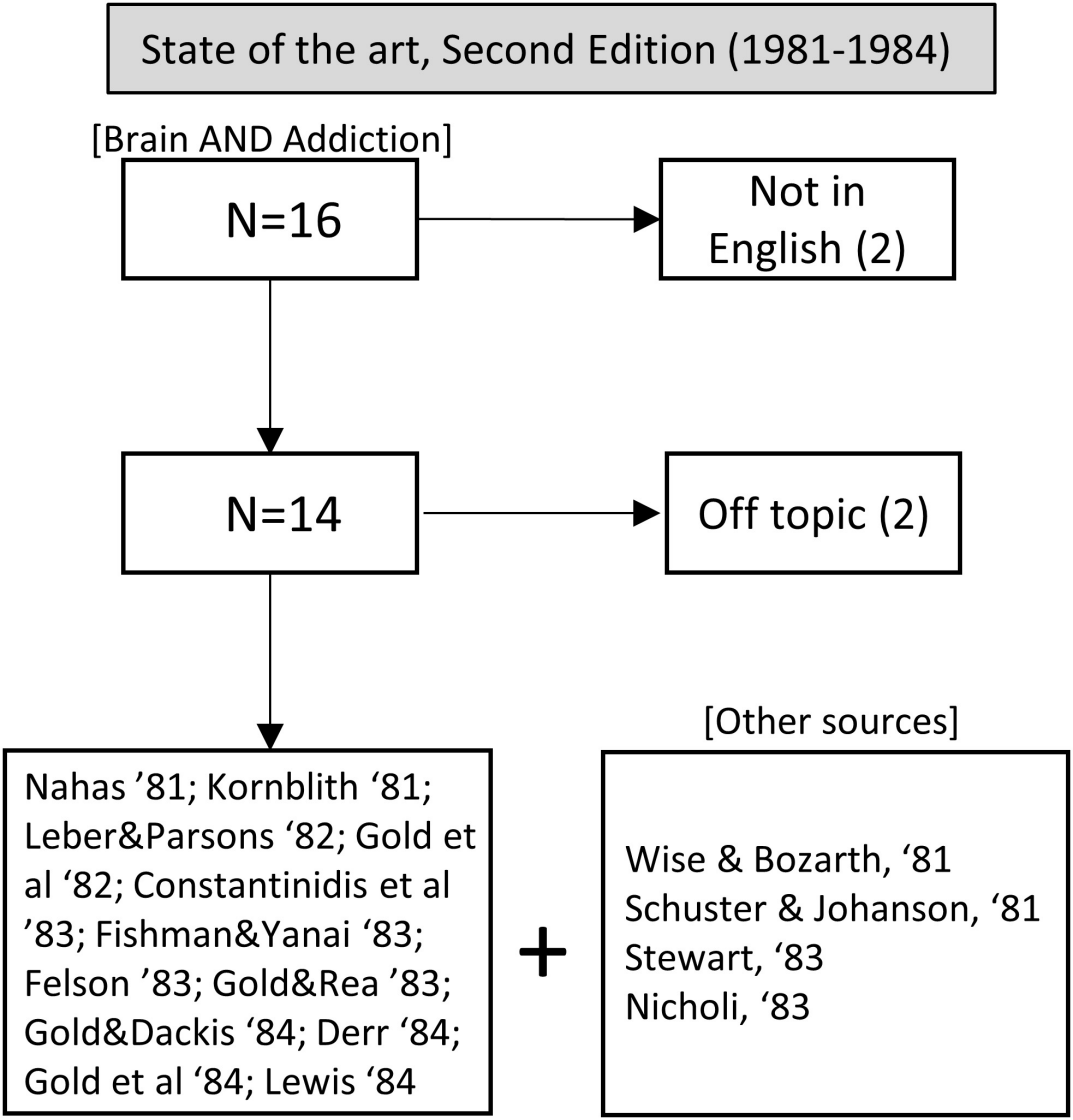
Flow chart showing article selection for the state of the art before the publication of the second edition of Kandel’s handbook.

During this period, we also adapted the search string to “brain” and “addiction” because the term “neurobiology” was not used. The output consisted of 16 articles. Two were excluded for not being in English, and two for being unrelated to addiction. The 12 selected articles were supplemented by other relevant sources from their bibliographies, for a total of 16 articles analyzed (see Figure 2).

Concerning frameworks and concepts, in the early 1980s, new terms to describe addiction gained popularity in scientific literature, and over time came to integrate the different psychological and neurobiological facets of addiction. The idea of addiction as a form of “neuroadaptation,” mentioned in studies of previous years (Goldstein, 1979), became popularized, and the neurobiological bases gained more importance. Where Solomon and Corbit described addictions as “diseases of adaptation” (Solomon and Corbit, 1978), researchers in later years began to integrate this neurobiological concept with behavioral aspects. Addiction, seen by some authors as a moral failing or psychological weakness (Nicholi, 1983), was consolidated as a compulsive behavior driven by neurobiological adaptations (Wise and Bozarth, 1981).

Nahas (1981) summarizes the “learning” theory for addiction, involving “pleasure reward mechanisms of the brain” which reinforce repeated drug-use behaviors. The main reinforcers are withdrawal symptoms and tolerance. Nahas questions the validity of distinguishing “psychic” and “physical” dependence, contradicting other researchers who argue in favor of this distinction, like Schuster and Johanson (1981), who introduce the term “craving” as the psychological or behavioral component of dependence. Stewart (1983) later mapped psychological dependence to a neurobiological cause –a neurobiological response to conditioned cues that activate brain reward pathways even in the absence of drugs.

Another influential theory that emerged during this period was the Psychomotor Stimulant Theory, proposed by Wise and Bozarth (1981). This theory is rooted in Wise’s Hedonic Hypothesis, published in the previous year, which suggested that addiction is a pleasure-seeking behavior reinforced by dopamine. This falls in line with some vulnerability-based hypotheses proposed in this period, such as Nicholi’s (1983), which states that patients take drugs to “escape from a less than tolerable reality” (Nicholi, 1983, p. 931).

Regarding specific substances and neural systems, Gold and colleagues contribute to a better understanding of opiate addiction in this time period, showing first the link between opiate addiction, endorphins and the possible treatment with naltrexone (Gold et al., 1982), then the role that endorphins play in addiction, withdrawal, and recovery (Gold and Rea, 1983), and finally the efficacy of different treatments for addiction (like clonidine) involving endogenous opioid peptides and hyperactive norepinephrine neurons (Gold et al., 1984; Gold and Dackis, 1984). Complementarily, Derr (1984) proposes a new hypothesis for ethanol withdrawal symptoms, suggesting that they result from decreased aerobic cell metabolism in the brain (an inability to properly conduct the Krebs cycle), and Constantinidis et al. (1983) reviews the role of different peptide neurotransmitters within the body, in the formation of a range of different neurological and psychiatric disorders, including addiction.

Lewis (1984), besides giving a neurobiological explanation of addiction, adds personality features as vulnerability factors. As such, he proposes that people with antisocial personality traits tend to present neurological alterations such as subcortical dysfunction, which could be linked to “deviant behavior.” All these factors would predate the development of addiction, and are proposed as a possible contributing factor.

Instead of exploring addiction itself, some articles focus on the general negative consequences that arise as a byproduct of addiction, including different substances such as alcohol (Leber and Parsons, 1982), non-opiate non-alcoholic substances (Kornblith, 1981), or barbiturates (Fishman and Yanai, 1983). A special issue in Seminars in Roentgenology is dedicated to the “Radiology of Drug Addiction,” describing the affectation of different organs due to this condition (Felson, 1983).

Overall, this period reflects a deeper debate on concepts such as adaptation, reward learning, craving and dependence, and the introduction of the psychomotor stimulant theory. More precise neurochemical studies center on opioids and endorphins, rather than on catecholamines. Also, several studies show the negative physiological effects of addiction, and stress the interplay between personality, neuroanatomy and addiction.

See Table 3 for a thematic summary of the literature during this time period.

**TABLE 3:** Key issues of the state of the art in the neurobiology of addiction before the publication of the second edition of Kandel’s handbook.

Issue	Main ideas	References
Frameworks and concepts	Adaptation/psychomotor stimulant theory	Wise and Bozarth, 1981
	Reward and learning	Nahas, 1981
	Craving	Schuster and Johanson, 1981
	Dependence	Stewart, 1983
Specific NT or biological systems	Endorphins	Gold et al., 1982; Gold and Rea, 1983
	Norepinephrine	Gold et al., 1984; Gold and Dackis, 1984
	Several neuropeptides	Constantinidis et al., 1983
Specific drugs	Opiates	Gold and Rea, 1983
	Ethanol	Derr, 1984
Vulnerability factors	Tolerance	Nicholi, 1983
	Personality	Lewis, 1984
Addiction effects	Negative physiological effects	Leber and Parsons, 1982; Kornblith, 1981; Fishman and Yanai, 1983; Felson, 1983

### 3.3 State-of-the-art in the third edition (1987–1990)

**FIGURE 3:**
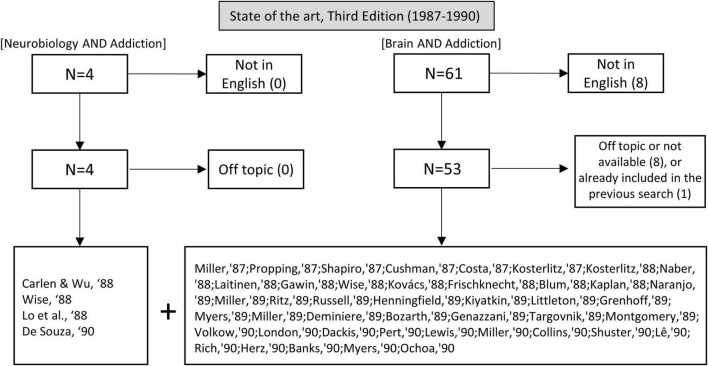
Flow chart showing article selection for the state of the art before the publication of the third edition of Kandel’s handbook.

In the second half of the 1980s, the word “neurobiology” started to be used in the context of addiction. The search “neurobiology” and “addiction” retrieved 4 articles. To complement this limited search, we repeated the query “brain” and “addiction,” yielding 61 additional articles. We excluded 16 articles for not being in English (8), being unrelated to addiction or drug use (5), or being inaccessible (3) (see Figure 3).

Interestingly, for both search strings, we did not find any article explicitly developing new or established frameworks in addiction. This fact was unique across all time periods. Concerning the first search (“neurobiology” and “addiction”), De Souza explains the effects of benzodiazepines in the neuroendocrine system, hardly referring to addiction (De Souza, 1990). The work by Lo and collaborators is on retroviral-mediated gene transfer and is far from addiction research (Lo et al., 1988). The two remaining papers do deal with addiction. First, Carlen and Wu (1988) explain the role of sedative-hypnotic drugs (such as alcohol, barbiturates, and benzodiazepines) on calcium-mediated metabolic processes, and their effect on tolerance, dependence, and withdrawal symptoms. Finally, Wise (1988) claims that theories rooted primarily in psychology were just descriptive, suggesting that psychological constructs such as the previously popularized “craving” were too subjective for addiction research. Instead, he suggests that psychological components should be measured through behaviors (according to them, preoccupation, compulsivity, and relapse), and should be integrated with the more robust neurochemical components, which are related to physiological dependence and tolerance (even if these are not necessary components for addiction).

Regarding the second search query (“brain” and “addiction”), included research articles explore addiction through neurobiological pathways, specific pharmacological mechanisms, and individual genetic or psychological vulnerabilities. A recurring theme across neurobiological pathways and reinforcement mechanisms is the role of the mesocorticolimbic dopaminergic pathway as a primary substrate for drug dependence. For example, cocaine reinforcement is directly linked to enhanced dopaminergic neurotransmission in the ventral tegmental system (Bozarth, 1989). Changes in this reward system, such as dopamine depletion, are hypothesized to drive the intense craving associated with stimulant addiction (Dackis and Gold, 1990). Concerning stimulants, Miller et al. provide an extensive review of amphetamines (Miller et al., 1989b). Other articles also explore the interplay between the dopaminergic system and other endogenous neuroactive substances. For example, Ochoa et al. (1990) highlight cholinergic mechanisms in nicotine addiction and explain in detail the molecular structure of nicotinic receptors. Myers (1990) links dopamine to enkephalin to support the “Multiple Metabolite” theory of alcoholism, which proposes the affectation of the limbic system by an endogenously formed aldehyde adduct: the neuronal damage it produces on dopaminergic and enkephalinergic pathways would be related to the rewarding and addictive properties of alcohol. In a previous work, Myers showed the role of tetrahydroisoquinoline and beta-carboline derivatives in increasing voluntary alcohol consumption by interacting with opioid receptors and the dopamine system (Myers, 1989).

Going deeper into neurochemical theories of alcohol use disorders, Blum and Trachtenberg (1988) propose alcoholism as a neuropsychogenetic disorder rooted in deficient endogenous opioids. According to this model, alcohol serves as an exogenous stimulator compensating for genetically or environmentally induced opioid deficiencies. They identify enkephalinase inhibitors as promising therapeutic tools. Another therapeutic avenue is directly modifying alcohol intake via serotonergic modulation. Naranjo and Sellers (1989) provide evidence from multiple placebo-controlled trials showing that serotonin reuptake inhibitors can reduce alcohol consumption by 20–30%, though without identifiable predictors of treatment response. In contrast, Cushman (1987) reviews ethanol–opioid interactions, concluding that metabolic and receptor-level interactions are complex and not yet clearly clinically interpretable. Naber (1988) also concludes that the link between endorphins and psychiatric/addictive disorders remains inadequately substantiated, reflecting uncertainty in translating opioid biology to treatment.

Further complicating the opioid landscape, another article identifies the possibility of additional opioid receptor subtypes, suggesting selective tolerance patterns and highlighting the μ-receptor’s clear association with high abuse potential (Herz, 1990). Additionally, several mechanistic biochemical papers, such as Kosterlitz et al. (1988) on cation modulation of opioid receptors, Kosterlitz (1987) on endogenous opioid ligands, and Costa et al. (1987) on opioid peptide biosynthesis, provide foundational molecular context relevant to many addiction models but do not propose new addiction-specific theories.

Concerning cocaine, extensively reviewed by Miller et al. (1989a), Ritz and colleagues demonstrate that self-administration correlates most strongly with its blockade of dopamine reuptake, not serotonin or norepinephrine (Ritz et al., 1989). Complementing this, Gawin (1988) shows that chronic cocaine use leads to neurophysiological adaptations reducing activity in reward circuits, resulting clinically in anhedonia during abstinence. Pert et al. (1990) link dopamine with the conditioning produced by psychomotor stimulants. Instead of relating dopamine to other neurobiological pathways, these authors highlight its role in the acquisition of conditioned behaviors characteristic of incentive-motivational processes.

Deminiere et al. suggested that increased dopamine turnover in the nucleus accumbens had a more widespread, non-specific role in drug-related behavior, also provoking a decreased dopamine turnover in the frontal cortex (Deminiere et al., 1989). Across substances, Wise (1988) proposes a two-factor reinforcement model: positive reinforcement through dopaminergic activation and negative reinforcement through suppression of distress signals, especially for opioids. This dual-system understanding becomes crucial for explaining craving and relapse independent of withdrawal symptoms. Nicotine addiction is also anchored in catecholaminergic mechanisms. Grenhoff and Svensson (1989) and Henningfield and Woodson (1989) document how nicotine influences dopamine and norepinephrine systems, reinforcing smoking behavior and producing dependence through dose-related neurochemical and behavioral changes.

Several studies concentrated on biological vulnerability, posing genetic influences as another significant theme: models using inbred strains, mutants, and selectively bred animals demonstrate strong heritable components in drug metabolism, receptor number, drug-seeking behavior, and withdrawal (Shuster, 1990). Frischknecht et al. (1988) compare opioid-reactive mouse strains, showing genetically determined dissociations between analgesia, locomotor response, and addiction vulnerability, and Collins shows that nicotinic receptor number is genetically regulated, reinforcing the view that addiction liability can be rooted in inherited neurobiological traits (Collins, 1990). Individual variability also shapes nicotine’s subjective effects. Russell (1989) discusses how innate factors, tolerance, receptor regulation, and learning produce highly variable nicotine responses in humans, influencing dependence trajectories. Genetic heterogeneity extends beyond opioids and nicotine. Propping (1987) argues that psychiatric disorders, including addictive propensities, likely arise from polygenic interactions, noting subtle differences even among heterozygotes for metabolic disorders.

Some studies expand the frame beyond neural mechanisms to cognitive and psychological dimensions. Neuropsychological research reports that substance abusers show deficits in abstract reasoning, cognitive flexibility, and behavioral control; these findings support the idea that “cognitive style” may serve as a bridge between neuropsychological functioning and personality-related addiction vulnerability (Miller, 1990). Others examine reinforcement mechanisms in alcohol addiction from both positive (euphoria, anxiolysis) and negative (withdrawal, aversion) reinforcement perspectives, integrating neurochemical and genetic findings into motivational models of alcohol use (Lewis, 1990). London et al. (1990) summarize findings about drug-induced euphoria using PET, being one of the first neuroimaging reviews. In the same issue of the NIDA Research Monograph series, Volkow et al. (1990) review PET studies about different drugs of abuse, such as cocaine, alcohol, and marijuana.

Another recurring topic is the biological basis of tolerance and withdrawal. Littleton (1989) proposes that chronic alcohol intake upregulates GABA function and modulates calcium channels, creating physical dependence and suggesting calcium-channel antagonists as a therapeutic avenue. More broadly, Kiyatkin (1989) reviews how chronic drug exposure induces stable modifications in central monoaminergic and opioid systems, altering endogenous reinforcement processes and solidifying dependence. Oxytocin emerges as a modulator of opioid tolerance and withdrawal. Kovács and Telegdy (1988) show that oxytocin reduces tolerance development and withdrawal symptoms in morphine- and heroin-treated animals, indicating a specific role in dependence but not analgesia. Expanding the concept of dependence, Miller et al. (1987) argue that addiction is not simply the presence of tolerance and withdrawal, which can develop even after a single dose, but rather a neurochemically driven distortion of instinctive drives. In their view, addiction may occur independently of classical dependence markers.

A subset of papers explores the physiological consequences of substance use rather than addiction mechanisms *per se*. Habitual smokers exhibit dose-dependent increases in plasma cortisol driven by nicotine’s central action on hypothalamic or brainstem structures, and smoking cessation decreases cortisol levels—a fluctuation linked to withdrawal symptoms (Targovnik, 1989). In another example, heroin addiction is associated with amenorrhea and hypogonadism, suggesting interference of endogenous opioid peptides with the hypothalamic-pituitary-gonadal axis (Genazzani and Petraglia, 1989). Also, Rich et al. (1990) describe a few cases of isopropyl alcohol intoxication, which can have different presentations in addicted (stupor) and non-addicted (encephalopathy) individuals. Similarly, Lê and Kalant (1990) explain how learning is a relevant factor in ethanol tolerance.

A few articles highlight broader neurobiological infrastructure relevant to addiction. The blood–brain barrier’s peptide transport systems are described as modulated by ethanol addiction and withdrawal, opening the possibility that such mechanisms influence peptide-based signaling relevant to dependence (Banks and Kastin, 1990). Another article describes the interplay between several serotonin receptors in psychiatric illnesses, briefly including addiction (Montgomery and Fineberg, 1989). Tangentially, albeit interestingly, Shapiro and Kornfeld (1987) highlight psychiatric considerations in patients with head and neck cancer, including high rates of alcohol and tobacco addiction: an important reminder of the intersection between addiction and severe medical illness. Laitinen explains neurosurgery in psychiatric disorders, including addiction, where cingulotomy is reported as effective (Laitinen, 1988). Finally, Kaplan proposes a provocative novel model linking drug experience, creativity, and cerebral lateralization. He suggests that psychoactive drugs initially enhance, then disrupt, creative processes by altering hemispheric balance, eventually producing disconnection patterns akin to split-brain phenomena (Kaplan, 1988).

In conclusion, addiction research during this period consolidated into a distinctly neurobiological framework, with “neurobiology” emerging as an explicit term and dopamine-centered models dominating explanations of reinforcement, craving, and relapse. The mesocorticolimbic pathway became the principal substrate for drug dependence across substances, while opioid, serotonergic, and cholinergic systems were integrated into increasingly complex, multi-neurotransmitter accounts. Genetic vulnerability, receptor regulation, and neurochemical adaptations were recognized as central to individual differences in addiction liability. At the same time, neuroimaging (PET), cognitive research, and studies of tolerance and withdrawal expanded the field’s methodological scope. Overall, addiction was increasingly conceptualized as a heritable, brain-based disorder shaped by neuroadaptation, learning, and motivational dynamics.

See Table 4 for a conceptual summary of this time period.

**TABLE 4:** Key issues of the state of the art in the neurobiology of addiction before the publication of the third edition of Kandel’s handbook.

Issue	Main ideas	References
Concepts	Behaviorist and neurochemical approach	Wise, 1988
	Tolerance and withdrawal	Littleton, 1989; Kiyatkin, 1989; Kovács and Telegdy, 1988
	Dependence	Miller et al., 1987
Specific NT or biological systems	Dopamine	Bozarth, 1989; Dackis and Gold, 1990; Ritz et al., 1989; Pert et al., 1990; Deminiere et al., 1989; Wise, 1988; Grenhoff and Svensson, 1989; Henningfield and Woodson, 1989
	Acetylcholine	Ochoa et al., 1990
	Dopamine + enkephalin	Myers, 1990
	Endogenous opioids	Blum and Trachtenberg, 1988; Naber, 1988
	Serotonin	Naranjo and Sellers, 1989; Montgomery and Fineberg, 1989
Specific drugs	Sedative/hypnotic	Carlen and Wu, 1988; Herz, 1990; Kosterlitz, 1987; Costa et al., 1987; Kosterlitz et al., 1988
	Cocaine	Bozarth, 1989; Miller et al., 1989a; Ritz et al., 1989; Gawin, 1988
	Amphetamines	Miller et al., 1989b
	Nicotine	Ochoa et al., 1990; Grenhoff and Svensson, 1989; Henningfield and Woodson, 1989
	Alcohol	Myers, 1989; Blum and Trachtenberg, 1988; Naranjo and Sellers, 1989
	Alcohol + opioids	Cushman, 1987
Vulnerability factors	Genetics	Shuster, 1990; Frischknecht et al., 1988; Collins, 1990
	Polygenic interactions	Propping, 1987
	Innate factors and others	Russell, 1989
Effects of addiction	Neuropsychological	Miller, 1990; Lê and Kalant, 1990
	Physiological	Targovnik, 1989; Genazzani and Petraglia, 1989; Rich et al., 1990
	Positive and negative reinforcement	Lewis, 1990; London et al., 1990
Other	Benzodiazepines and neuroendocrine	De Souza, 1990
	Retroviral gene transfer	Lo et al., 1988
	Neuroimaging studies (PET)	London et al., 1990; Volkow et al., 1990
	Blood-Brain Barrier	Banks and Kastin, 1990
	Psychiatry in head and neck cancer	Shapiro and Kornfeld, 1987
	Neurosurgery in psychiatry	Laitinen, 1988
	Drugs, creativity and lateralization	Kaplan, 1988

### 3.4 State-of-the-art in the fourth edition (1996–1999)

**FIGURE 4:**
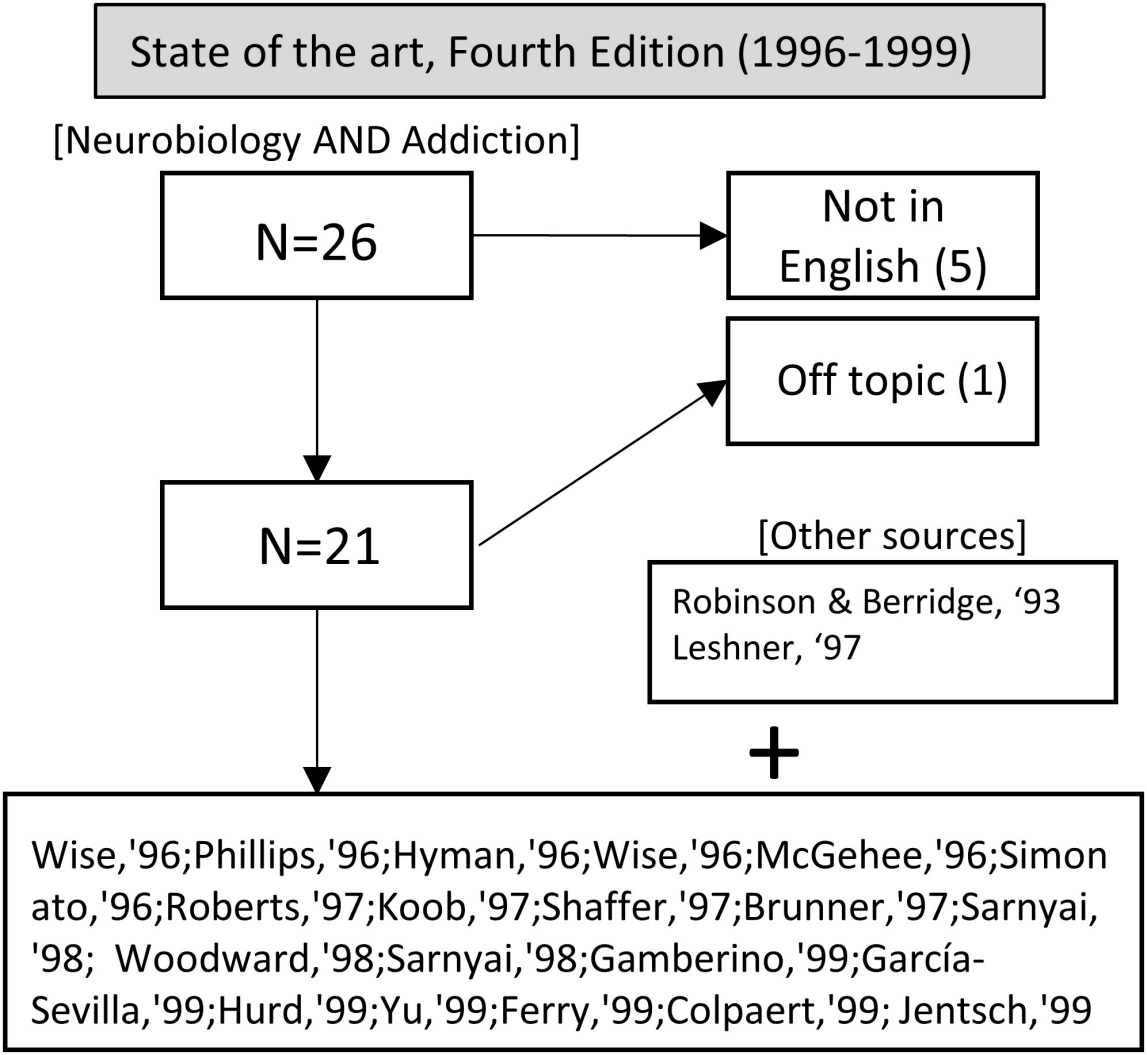
Flow chart showing article selection for the state of the art before the publication of the fourth edition of Kandel’s handbook.

The query “neurobiology” and “addiction” yielded 26 articles published between 1996 and 1999. Five were excluded because they were not in English, and 1 reference was not about addiction. We also included two more references for their outstanding importance in the neurobiology of addiction: Robinson and Berridge (1993) and Leshner (1997). The former did not result in the original search because it was published before 1996, and the latter was indexed in PubMed as a journal article rather than a review, editorial, or any other article type originally included in our search queries (see section “1 Introduction”; see Figure 4).

In this period, addiction research grew increasingly integrative, blending neurobiological, behavioral, molecular, and conceptual insights. Before diving into specific frameworks, several authors address the fundamental nature of addiction. Koob and Nestler give a good summary of these related aspects in their 1997 review; drug addiction or dependence is defined as “a compulsion to take a drug, with a loss of control in limiting intake” (Koob and Nestler, 1997, p. 482). Reinforcement or motivation is considered a crucial part of this syndrome, where a reinforcer is defined as “any event that increases the probability of a response” (p. 483). Reward is similar, but specifically includes pleasure. The components of addiction are pleasure, self-medication, and habits, both as conditioned positive and negative reinforcement. These aspects are reflected for the first time in the 4th edition of Kandel’s *Principles*, which is the first of the six editions to give proper consideration to the topic of addiction. Remarkably, Leshner overtly presents drug addiction as a brain disease, defining it as a chronic, relapsing disease characterized by compulsive drug seeking (Leshner, 1997). More importantly, while highlighting the role of the brain in the disease, the author insists that it is not just a brain disease, so therapeutic approaches must consider biological, behavioral, and social-contextual components. The psychomotor stimulant theory remained a significant focus in the literature of these years (Koob and Nestler, 1997; Sarnyai, 1998a; Wise, 1996). Wise speaks of recent contributions to the theory, namely, the central nervous system’s newfound role in “mediating positive reinforcement and euphoria” (Wise, 1996, p. 243). He also shows evidence against negative reinforcement (such as withdrawal symptoms) as the motor of drug abuse, stating that “human alcoholics were shown to voluntarily eschew alcohol during periods of severe withdrawal distress (only to re-initiate drinking when such distress was minimal)” (Wise, 1996, p. 244). Additionally, Sarnyai gives a “stress version” of psychomotor stimulant theory rather than a “reward version,” as he explains that all the effects of cocaine (behavioral hyperactivity, euphoria, addiction per se, withdrawal, etc.) are explained by its impact on the stress circuit via the corticotropin releasing factor (Sarnyai, 1998a).

An important new theory that appears in the literature is Incentive Salience Theory, proposed by Robinson and Berridge (1993), which shifts away from the notions of pleasure-seeking emphasized by the popular psychomotor stimulant theory across previous decades (Robinson and Berridge, 1993). This hypothesis explains addiction as a transition from “liking” to “wanting” the drug (another term for the previously discussed “craving”), which is explained by “progressive and persistent neuroadaptations” due to persistent drug use. This implies a change from goal-directed and voluntary actions to compulsive self-administration despite liking the drug much less than at the initial encounter, which is a concept that will be further developed in the following years.

Amidst this variety of frameworks, one perspective highlights a state of “conceptual chaos,” arguing for clearer definitions of substance use, abuse, and dependence, while suggesting that drugs may not be a necessary precondition for addiction, as seen in behaviors like gambling (Shaffer, 1997). Others define addiction broadly as a compulsion to use drugs and the experience of withdrawal, a state involving both physical hyperexcitability and mental shifts that motivate relapse (Roberts and Koob, 1997). Overall, a key message is that while different drugs like heroin and cocaine have specific “chemical trigger zones,” they ultimately converge on the same shared brain mechanisms of reward (Wise, 1996). Colpaert (1999) proposes a new framework to understand addiction, namely drug discrimination studies, as determinants of areas of neurobiological interest (ligand analysis, CNS receptors, enzymes, ion channels). This is conceived as a quantal assessment of drug mechanisms focused on molecular effects. On their side, Hyman and Nestler review the state of the art in addiction neurobiology to promote a paradigm shift from synapses to the molecular biology of neurons beyond interneuronal communication (Hyman and Nestler, 1996). They describe how neural plasticity occurs from the start to its final adaptation after long-term exposure to drugs, including gene expression.

Moving to specific neurobiological systems, and expanding the interest in dopamine of previous years, a dominant theme across several articles is the central role of frontostriatal and mesocorticolimbic circuits in shaping compulsive drug-seeking behavior. For instance, Jentsch and Taylor (1999) highlight how prolonged drug exposure may impair frontal cortical regions required for inhibitory control, contributing to compulsive reward-driven actions. Chronic multineuron recording techniques allow researchers to observe these neuronal behaviors in real-time within the mesocorticolimbic circuit during active drug use (Woodward et al., 1998). Further, ethanol’s effects on locomotion are traced back to the dopaminergic pathways between the ventral tegmental area and the nucleus accumbens (Phillips and Shen, 1996). More broadly, Gamberino and Gold emphasize that most drugs of abuse converge on dopamine-rich pathways that mediate reinforcement, learning, and sensitization (Gamberino and Gold, 1999).

Regarding specific substances, cocaine and stimulant-related studies form another major cluster. Hurd and colleagues outline how cocaine disrupts dopamine, dynorphin, and a specific peptide (the cocaine- and amphetamine-regulated transcript, CART) signaling within the ventral striatum and amygdala, supported by in vivo microdialysis and in situ hybridization (Hurd et al., 1999). A complementary review shows that oxytocin and vasopressin modulate cocaine tolerance, dependence, and sensitization across basal forebrain, hippocampal, hypothalamic, and limbic systems, with particular interactions in the nucleus accumbens (Sarnyai, 1998b). This is linked to biological vulnerability to addiction. Imidazoline receptors also play a role, in this case on opioid addiction; their ligands can reduce opioid tolerance, yet these receptors are notably absent in the brains of heroin and morphine addicts (García-Sevilla et al., 1999). Remarkably, Simonato (1996) frames morphine addiction as a product of complex, nonlinear interactions in distinct neocortical neuronal populations, shaped by chronic opioid exposure and downstream second-messenger systems. Finally, and related again to biological vulnerability, Brunner and Hen (1997) carry out genetic studies using serotonin receptor knockout mice to demonstrate that a lack of 5-HT1B receptors leads to stronger impulsive behaviors and an increased tendency toward addiction.

Tangentially related to addiction, McGehee and Role (1996) describe presynaptic ionotropic receptors gated by GABA, nicotine and glutamate, and state their potential role in addiction. In a similar way, Yu and Salter (1999) discuss the neurobiology of NMDA receptors, with no mention to addiction. Ferry (1999) focuses on pharmacological treatment for smoking cessation.

In conclusion, addiction research during this period entered a phase of conceptual refinement and molecular expansion, consolidating prior dopaminergic models while integrating emerging insights from genetics, neuroplasticity, and systems neuroscience. Addiction was increasingly defined as compulsive drug use driven by reinforcement, habit formation, and enduring neuroadaptations, with influential frameworks such as Incentive Salience Theory shifting emphasis from pleasure (“liking”) to pathological “wanting.” Also, a behavioral addiction such as gambling is mentioned for the first time in this context. At the same time, advances in molecular biology, gene expression, and circuit-level recording deepened understanding of frontostriatal and mesocorticolimbic dysfunction in compulsivity.

See Table 5 for the conceptual summary of this time period.

**TABLE 5:** Key issues of the state of the art in the neurobiology of addiction before the publication of the fourth edition of Kandel’s handbook.

Issue	Main ideas	References
Frameworks and concepts	General overview	Koob and Nestler, 1997
	Psychomotor stimulant theory	Koob and Nestler, 1997; Wise, 1996
	Idem, stress version	Sarnyai, 1998a
	Incentive salience	Robinson and Berridge, 1993
	Compulsion and withdrawal	Roberts and Koob, 1997
	Drug discrimination studies	Colpaert, 1999
	Molecular biology	Hyman and Nestler, 1996
	Conceptual chaos	Shaffer, 1997
Specific NT or biological systems	Dopamine	Jentsch and Taylor, 1999; Woodward et al., 1998; Phillips and Shen, 1996; Gamberino and Gold, 1999
	Dopamine + neuropeptides	Hurd et al., 1999
	Dopamine + oxytocin/vasopressin	Sarnyai, 1998b
Specific drugs	Cocaine	Hurd et al., 1999; Sarnyai, 1998b
	Opioids	García-Sevilla et al., 1999; Simonato, 1996
	Alcohol	Phillips and Shen, 1996
Vulnerability factors	Genetics	Brunner and Hen, 1997
	Neurochemistry	Sarnyai, 1998b; García-Sevilla et al., 1999
Other	Presynaptic ionotropic receptors	Yu and Salter, 1999
	Treatment for smoking	Ferry, 1999

### 3.5 State-of-the-art in the fifth edition (2008–2011)

**FIGURE 5:**
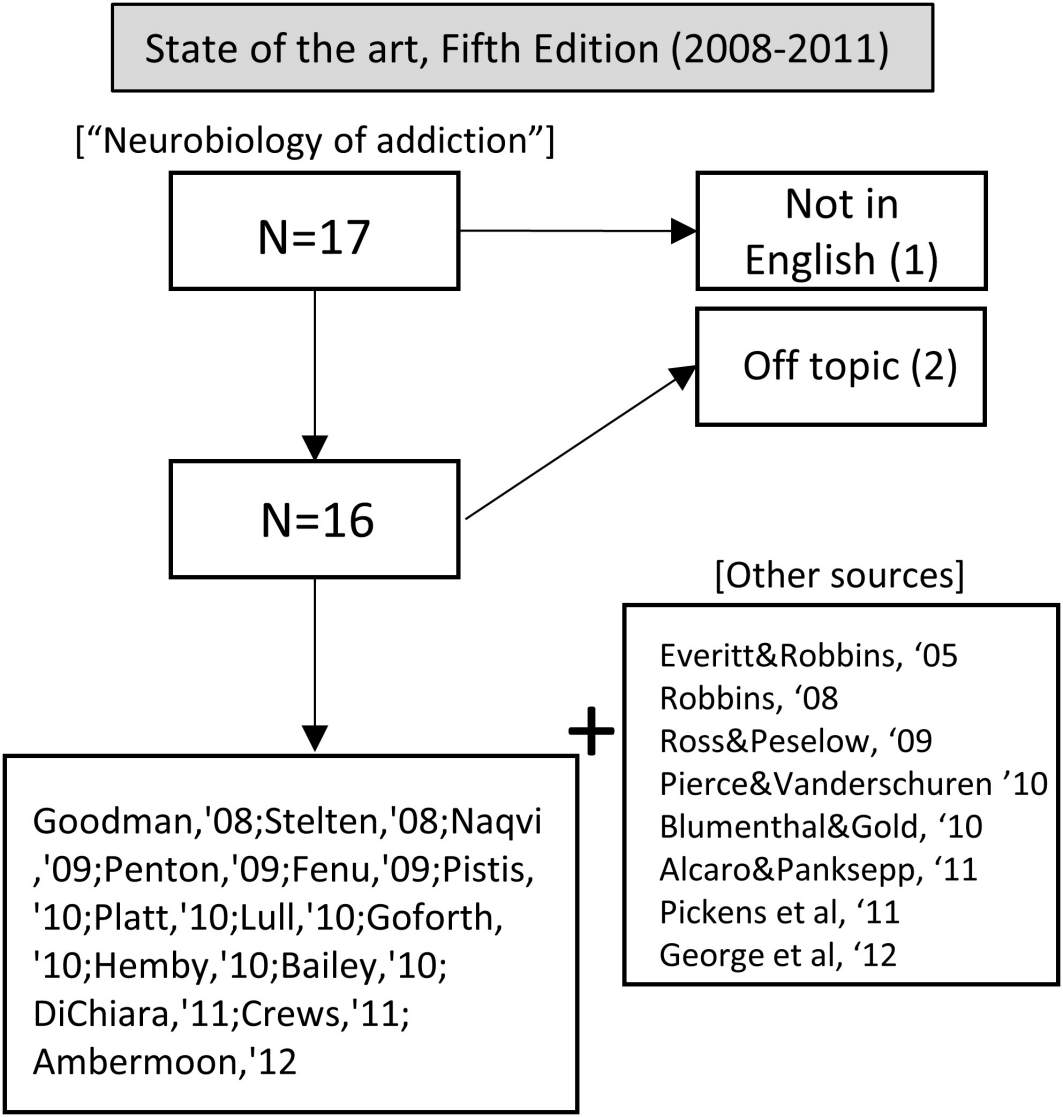
Flow chart showing article selection for the state of the art before the publication of the fifth edition of Kandel’s handbook.

In these years, we used the term “neurobiology of addiction” in our query, yielding 17 articles; one was excluded because it was not in English, and 2 were unrelated to addiction. Also, we incorporated other relevant sources from the bibliographies of the selected articles (see Figure 5).

An important factor incorporated in the definition of addiction during these years is that of relapse, defining addiction as a “chronically relapsing disorder” (George et al., 2012, p. 59). Another revived idea is that of addiction as an illness: according to Ross and Peselow, it is a “neurobiological illness” involving the “corruption” of the normal reward circuitry (Ross and Peselow, 2009). There is also a greater emphasis on the role of memory in defining addiction. Robbins and collaborators framed addiction as a pathological learning and memory disorder in which drug-related memories override normal cognitive functions (Robbins et al., 2008), suggesting another possible contributor to the previously mentioned concept of drugs as usurpers of normal drive states or instincts (Miller et al., 1987).

Precisely, Everitt and Robbins proposed a new theory of addiction in 2005, where there is a shift from goal-directed actions to rigid and ateleological habits to compulsions (Everitt and Robbins, 2005). This theory explains the progressive loss of control of the addicted person over their actions as they become more automatic and compulsive through repeated drug use (see also Robbins et al., 2008). Additionally, this is anatomically attributed to a transition from activity in the ventral striatum to the dorsal striatum. Pierce and Vanderschuren later explain this in terms of simultaneous behavioral plasticity, suggesting that behavior becomes progressively inflexible throughout the transition to stimulus-response actions, though it need not be irreversible (Pierce and Vanderschuren, 2010). Alcaro and Panksepp (2011) also defined the construct of drug-seeking as an overt behavioral response to self-administer the drug, including memory and cognitive effects, and positive affective states related to drug use. Another interesting idea is that of the “incubation” of drug craving, coined in 1986, in which craving progressively increases following abstinence after drug use, occurring over the course of weeks (Pickens et al., 2011). This is attributed to an increased risk of relapse.

George et al. (2012) propose an alternative opponent process theory, based on neurobiology, to explain allostasis (the flexible adaptation to maintain balance through physiological or behavioral change) in drug addiction. According to these authors, there are twofold opponent processes in this condition: one within a neurobiological system (namely, the dopaminergic system) and one between systems (affecting the corticotropin-releasing factor system).

During this period, the neural connections between known brain regions were significantly refined, resulting in more definitive neural circuitry. For example, Pickens et al. suggested that the nucleus accumbens, specifically its shell, may be connected to the full network of descending neural influences on reflexive autonomic and motor responses (locomotion) associated with drug use (Pickens et al., 2011). They also suggested that the nucleus accumbens shell may have dopaminergic projections to the ventral tegmental area, accumbens core, and ventromedial prefrontal cortex, involved in stress-related mechanisms and craving in cocaine addiction. Apart from these “traditional” brain regions, the insula is highlighted as a “hidden island” that integrates bodily signals into conscious urges, directly provoking relapse (Naqvi and Bechara, 2009).

At a neurotransmitter level, there was an integration of dopaminergic circuits with other systems, such as glutamatergic. Ross and Peselow explain that a neurochemical shift occurs from a more “dopamine-based behavioral system to a predominantly glutamate-based one,” (Ross and Peselow, 2009, p. 269) caused by later dysregulated glutamate transmission in the prefrontal cortex and nucleus accumbens. About dopamine, the compulsive use of dopamine replacement therapy in Parkinson’s patients is proposed as a novel model for understanding the neurobiology of stimulant addiction (Ambermoon et al., 2012). This is linked to studies on impulse control disorders in the same patient population, which identify dopamine D3 receptors and sensitization as key drivers of addictive behavior (Fenu et al., 2009). At the receptor level, 5-HT6 receptors in the ventral striatum are found to influence reward and reinforcement indirectly by modulating dopamine transmission (Di Chiara et al., 2011).

This time period also reveals a variety of topics in addiction research. A significant portion of the research delves into the “omics” and cellular signaling. Hemby (2010) explores genomic and proteomic changes in the brain to understand the molecular basis of cocaine abuse, while Lull et al. (2010) present neuroproteomics as a “stepping stone” for identifying new therapeutic targets and refining animal models. Innovative theories also link the innate immune system to addiction, suggesting that drug use triggers NF-kappaB transcription of proinflammatory mediators, which leads to a loss of hippocampal neurogenesis and increased negative emotions (Crews et al., 2011). Other molecular studies examine how nicotine regulates acetylcholine receptor subtypes (Penton and Lester, 2009), and how endocannabinoid ligands binding to nuclear receptors provide anti-addicting properties (Pistis and Melis, 2010).

Furthermore, a broad review argues that substance abuse and behavioral addictions (like gambling and bulimia) share common physiological processes involving motivation and reward, affect regulation, and behavioral inhibition (Goodman, 2008). The emerging field of neuroeconomics is also introduced to explain how the brain calculates the value of rewards and punishments under social or uncertain conditions (Platt et al., 2010), and its relevance to understanding addiction. Researchers also emphasize the necessity of integrated training because chronic pain and addiction frequently coexist, requiring specialized management strategies (Bailey et al., 2010). With the development of functional magnetic resonance imaging (fMRI), research paradigms in this period expanded from mostly animal research to neuroimaging studies in humans. For example, Blumenthal and Gold cite MRI evidence supporting the similarities between the anatomical regions involved in food addiction and classic substance addictions (Blumenthal and Gold, 2010). Finally, regarding treatments, general overviews provide a foundation on the neurologic effects of various drugs and current treatment options (Goforth et al., 2010). Looking toward future neurological interventions, research identifies deep brain stimulation as a potential neurosurgical treatment for severe addiction (Stelten et al., 2008).

In conclusion, during this period, addiction was firmly conceptualized as a chronic, relapsing neurobiological illness characterized by maladaptive learning, compulsive habits, and progressive loss of control. Theoretical advances emphasized the transition from goal-directed drug use to rigid stimulus–response habits, supported by refined models of ventral-to-dorsal striatal circuitry and allostatic dysregulation involving dopaminergic, glutamatergic, and stress systems. Relapse, craving incubation, and the insula’s role in interoceptive urges became central constructs. Simultaneously, molecular and “omics” approaches, immune signaling, receptor-level adaptations, and neuroimaging in humans expanded the field’s scope. Addiction research thus evolved into a highly integrative, circuit-based and translational neuroscience, linking behavior, biology, and emerging therapeutic interventions.

See Table 6 for a summary of the topics covered in this time period.

**TABLE 6:** Key issues of the state of the art in the neurobiology of addiction before the publication of the fifth edition of Kandel’s handbook.

Issues	Main ideas	References
Frameworks and concepts	Allostasis	George et al., 2012
	Neurobiological illness	Ross and Peselow, 2009
	Habits and compulsions	Everitt and Robbins, 2005; Robbins et al., 2008
	Inflexible behavior	Pierce and Vanderschuren, 2010
	Drug seeking	Alcaro and Panksepp, 2011
	Incubation of craving	Pickens et al., 2011
Specific NT or biological systems	Accumbens and its projections	Pickens et al., 2011
	Insula	Naqvi and Bechara, 2009
	Dopamine	Ambermoon et al., 2012; Fenu et al., 2009
	Dopamine to glutamate	Ross and Peselow, 2009
	Acelycholine receptors	Penton and Lester, 2009
	Serotonin receptors	Di Chiara et al., 2011
Novel methods	“Omics”	Hemby, 2010; Lull et al., 2010; Crews et al., 2011; Penton and Lester, 2009; Pistis and Melis, 2010
	Neuroeconomics	Platt et al., 2010
	fMRI	Blumenthal and Gold, 2010
Vulnerability factors	Chronic pain and addiction	Bailey et al., 2010
Other	Neurologic effects and treatment	Goforth et al., 2010
	Deep brain stimulation	Stelten et al., 2008
	Behavioral addictions	Goodman, 2008

### 3.6 State-of-the-art in the sixth edition (2017–2020)

**FIGURE 6:**
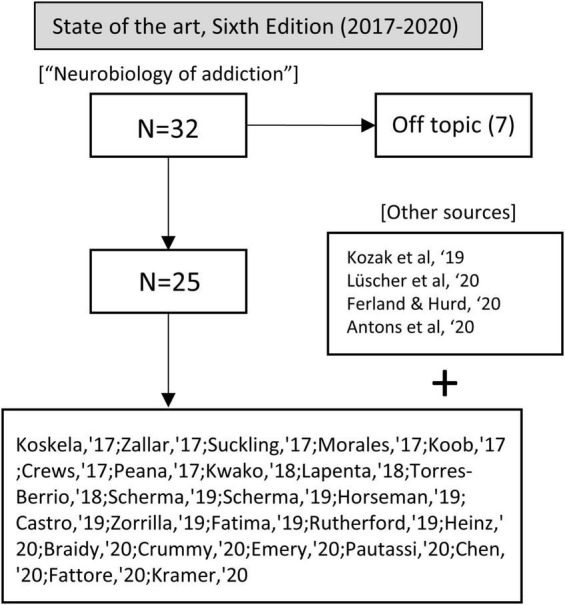
Flow chart showing article selection for the state of the art before the publication of the sixth edition of Kandel’s handbook.

The search query “neurobiology of addiction” for this time period resulted in 32 articles, 7 of which were excluded for being unrelated to addiction. They were supplemented with other contributions from their bibliographies (see Figure 6).

There is a convergence on a multi-system account of addiction that integrates motivational theory, circuit and neuromodulator mechanisms, and translational biomarkers. Conceptually, several papers emphasize that addiction cannot be reduced to tolerance and withdrawal alone; instead, compulsive seeking, craving, and a shared neurobiological substrate differentiate addictions from mere physiological dependence (e.g., caffeine, antidepressants). This perspective aligns with allostasis and incentive–sensitization frameworks and explicitly argues that later stages pivot toward negative reinforcement (Heinz et al., 2020; Horseman and Meyer, 2019; Koob, 2017; Kramer et al., 2020). Years after the original publishing of his theory, Koob wrote in 2020 about his version of opponent process theory, integrating aspects of stress and allostatic load (Koob, 2020). He now describes the dysphoric State B in terms of hyperkatifeia, a strong stress-induced negative emotional state. As in previous years, some conflated definitions have been clarified here as well. Lüscher et al. made the distinction between drug-seeking behaviors (the psychological or affective component associated with incentive states) and drug-taking behaviors (the measurable behavior of compulsive drug self-administration) (Lüscher et al., 2020). Kozak et al. also refined the difference between impulsivity and compulsivity, being the former a vulnerability marker for addiction and possibly an intrinsic personality trait associated with drug-seeking behaviors (Kozak et al., 2019).

Within this theoretical scaffold, dopamine system control emerges as a key mechanistic hinge. Failures in presynaptic dopamine D2 receptors increase addiction liability, with knockout/mutant models mapping how the interactions of these receptors reorganize reward circuitry and gating of reinforcement signals (Chen et al., 2020). Complementing this, circuit level reviews of the ventral tegmental area and nucleus accumbens synthesize how heterogeneous neuronal populations and neuropeptidergic inputs shape motivational states, including transitions from controlled use to compulsion (Castro and Bruchas, 2019; Morales and Margolis, 2017). Apart from dopamine, Emery and Akil discuss the dysregulation of the endogenous opioid system as a marker for opioid addiction and mood disorders (Emery and Akil, 2020). Further, the work by Scherma and colleagues demonstrates the role of anandamide, a lipid mediator, as a broad modulator of reward, capable of potentiating drug effects and biasing valuation in mesocorticolimbic loops (Scherma et al., 2019b). In another contribution, these authors review maladaptive eating habits in animal models (Scherma et al., 2019a).

Apart from dopamine and its interaction with other neurotransmitter systems, a complementary biological axis is neuroimmune signaling. One review centers on Toll-like receptors and microglia, arguing that drugs of abuse, as well as alcohol, enhance microglia activation through this pathway, linking inflammatory tone to addiction stage transitions (Crews et al., 2017). Closely related, several entries position stress as both precipitant and amplifier of addictive behavior, spanning negative urgency and motivational dysregulation, while also analyzing this issue in broader sociocultural contexts (Torres-Berrio et al., 2018; Zorrilla and Koob, 2019). Other research pinpointed the role of neurotrophic factors: Koskela et al. (2017) present addiction as loss of control over drug use with high rates of relapse, and report that neurotrophic factors BDNF and GDNF increase craving after drug self-administration. Additionally, Peana and colleagues review the role of acetaldehyde in alcoholism, showing its role in different stages of ethanol self-administration (Peana et al., 2017). About alcoholism, Pautassi et al.’s editorial discusses the association between early and late use of alcohol (Pautassi et al., 2020). Again related to stress, and concerning vulnerabilities, other articles highlight developmental and family context vulnerabilities, including the proposal that parenting stress constitutes a novel pathway to addiction risk, particularly around prenatal/postpartum windows when stress–reward interactions are dynamically remodeling caregiving and motivation circuits (Rutherford and Mayes, 2019).

About specific substances, as a response to the sociopolitical discussion surrounding marijuana legalization in recent years, where cannabis has been popularly considered a “soft,” less addictive drug, Ferland and Hurd state the potential dangers of cannabis use disorder (Ferland and Hurd, 2020). They argue that cannabis involves the same neuroadaptations and subsequent associated risks as other substance use disorders. Two additional translational themes surface. First, polysubstance use is common: Crummy et al. (2020) report that 11.3% of persons diagnosed with substance-use disorder have alcohol plus other substance dependence. Second, Kwako and collaborators introduce the Addiction Neuroclinical Assessment to identify novel biomarkers and refine current ones. For example, “reinforcer pathology” appears as an important new biomarker (Kwako et al., 2018). The literature also flags sex differences as evident, especially for novel psychoactive substances, but still insufficiently explained mechanistically across animal and human data, supporting sex informed designs in both preclinical and clinical work (Fattore et al., 2020). On the therapeutic horizon, mechanistic reviews propose targeting metabolic and hormonal systems: raising NAD^+^ as a putative strategy to treat addiction (Braidy et al., 2020), and ghrelin signaling as a candidate node linking stress and reward, with suggestive evidence across alcohol and stimulants (Zallar et al., 2017). Other authors also review the use of brain stimulation as a potential tool to alleviate addiction and impulse disorders (Lapenta et al., 2018).

Methodologically, one through line is human neuroimaging. Suckling and Nestor report frontostriatal disturbances across cognitive domains in users, some predictive of relapse and treatment response. They also find white matter changes in the anterior aspects of the brain, and suggest the role of cerebral vasculature in these processes (Suckling and Nestor, 2017). Focused imaging work on cannabis use disorder similarly maps alterations in reward, control, and decision-making networks with fMRI, illustrating how domain-specific markers could stratify risk and prognosis (Fatima et al., 2019).

Finally, a remarkable emerging topic is behavioral addiction, officially termed Impulse Control Disorders, which is still in its earliest stages of discussion. Antons et al. explain that different impulse control disorders seem to trigger different aspects of the known neural circuitry associated with substance use disorders; for example, whereas gaming disorder and compulsive sexual behavior disorder have been associated with altered activity in the salience network, gambling disorder has not (Antons et al., 2020). Hence, they call for longitudinal neurobiological studies to examine the later stages of impulsive control disorders and fully understand their development, as there is a general lack of research to explain such differences.

In conclusion, in this most recent period, addiction research converges on a fully integrative, multi-system model that links motivational theory, circuit dynamics, neuromodulators, immune signaling, and translational biomarkers. Addiction is clearly distinguished from mere physiological dependence, defined instead by compulsive seeking, craving, relapse vulnerability, and shared neurobiological substrates shaped by allostasis, stress, and incentive sensitization. Dopaminergic, opioid, endocannabinoid, and neuroimmune mechanisms interact within refined circuit models of frontostriatal and mesolimbic dysfunction, while neuroimaging and biomarker initiatives strengthen clinical translation.

Table 7 summarizes the main concepts and findings included in this time period.

**TABLE 7:** Key issues of the state of the art in the neurobiology of addiction before the publication of the sixth edition of Kandel’s handbook.

Issues	Main ideas	References
Frameworks and concepts	Addiction vs. physiological dependence	Heinz et al., 2020; Horseman and Meyer, 2019; Koob, 2017; Kramer et al., 2020
	Opponent process, stress and allostasis	Koob, 2020
	Drug seeking vs. taking	Lüscher et al., 2020
	Impulsivity vs. compulsivity	Kozak et al., 2019
Specific NT or biological systems	Dopamine, D2 receptors	Chen et al., 2020
	Dopamine, anandamide	Scherma et al., 2019b
	Accumbens, Ventral tegmental area	Castro and Bruchas, 2019; Morales and Margolis, 2017
	Endogenous opioids	Emery and Akil, 2020
	Neuroimmune system	Crews et al., 2017
	Stress	Torres-Berrio et al., 2018; Zorrilla and Koob, 2019
	Neurotrophic factors	Koskela et al., 2017
Specific drugs or behaviors	Alcohol	Peana et al., 2017; Pautassi et al., 2020
	Cannabis	Ferland and Hurd, 2020; Fatima et al., 2019
	Behavioral (Impulse Control Disorders)	Antons et al., 2020; Scherma et al., 2019a (animal models)
Vulnerability factors	Parenting stress	Rutherford and Mayes, 2019
	Sex differences	Fattore et al., 2020
Other	Polysubstance use	Crummy et al., 2020
	Addiction Neuroclinical Assessment	Kwako et al., 2018
	Therapeutic approaches	Braidy et al., 2020; Zallar et al., 2017; Lapenta et al., 2018
	fMRI review	Suckling and Nestor, 2017

## 4 Discussion

**FIGURE 7:**
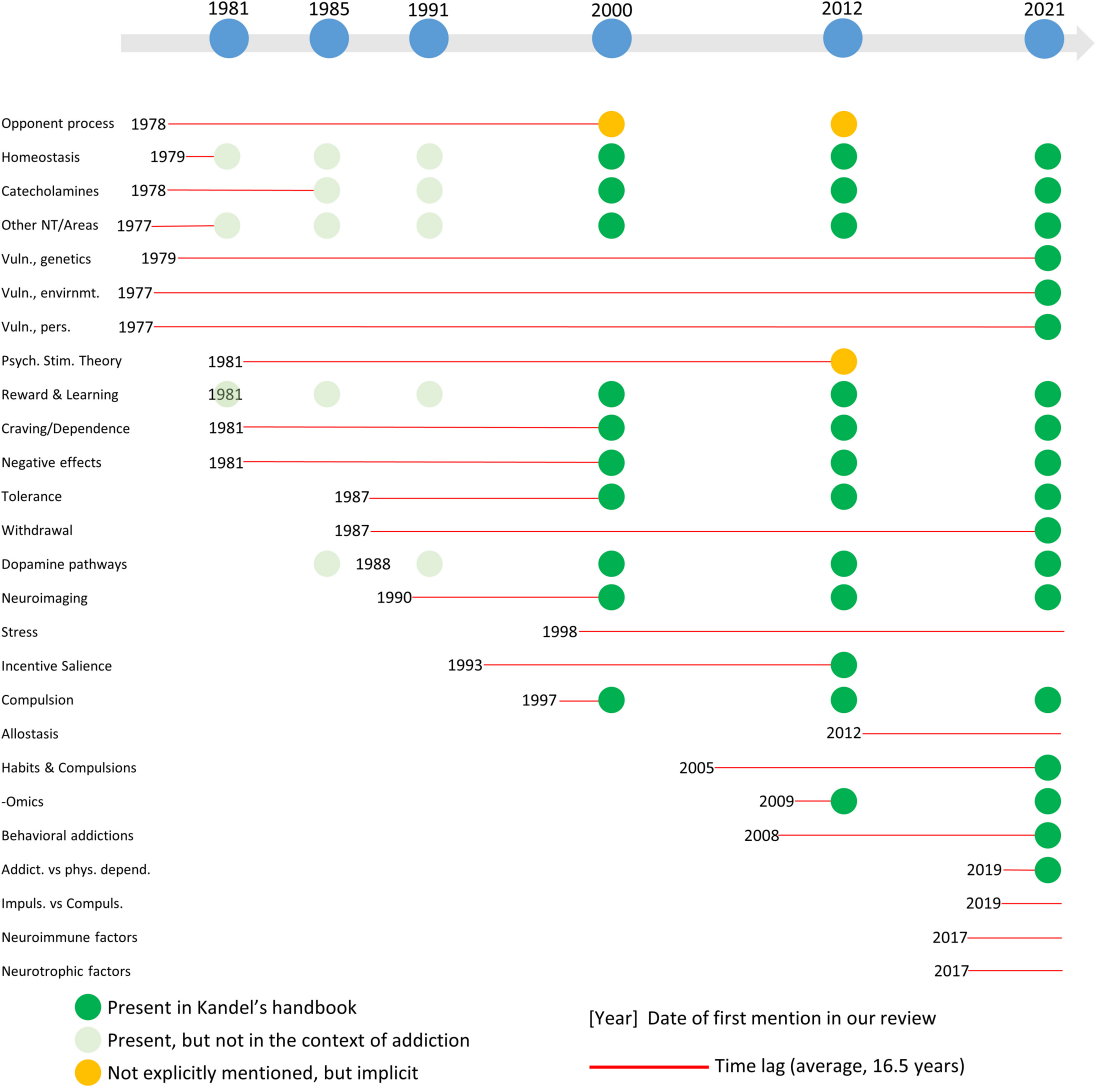
Visual comparison between the inception of the main concepts in the literature and their appearance in Kandel’s handbook. Blue circles indicate the publication of an edition of Kandel’s handbook. Years indicate when a concept appeared in the scientific literature according to our review. Dark green circles indicate when the concept was included in Kandel’s handbook. Light green circles mean that the topic appears in the handbook, but in a context unrelated to addiction. Yellow circles indicate that the theory is not explicitly mentioned in the textbook, but it is implicitly assumed. Red lines show the time lag between the introduction of the concept in the scientific literature and in Kandel’s handbook (average time lag for all concepts, 16.5 years). Note two odd cases: first, the concept “Reward and Learning” apparently is included in the scientific literature and the handbook in the same year. However, the state of the art already discussed reward/pleasure within the opponent process theory. Second, “Dopamine pathways” seems to appear first in the handbook and then in the literature. However, the role of dopamine had already been consolidated in scientific reviews under the concept “Catecholamines.” Addict. Vs. phys. depend, addiction versus physiological dependence; Impuls. Vs. Compuls, impulsivity versus compulsivity; NT, neurotransmitters; pers, personality; Psych. Stim. Theory, psychomotor stimulant theory; Vuln, vulnerability.

Our analysis reveals how the neurobiology of addiction has evolved over the last four decades and how this evolution has been explained in the most widely recognized handbook of neural science, Kandel’s *Principles of Neural Science*. The idea of comparing the *Principles’* content with the state of the art of each time period is not to provoke a confrontation or to expose the limitations of the manual, but to show whether there is a balance in the various psychological and neurobiological hypotheses of addiction as they are introduced to the neuroscientific community, or whether this information is biased toward specific perspectives. This is intended to be not just retrospective, but it could be useful to inspire how certain topics (for example mental disorders, but also other research issues) should be presented to the neuroscience community. See Figure 7 for a visual comparison between the inception of an addiction-relevant topic in the literature, and its appearance in Kandel’s handbook. On average, the time lag (inception in the literature subtracted from the year of appearance in Kandel’s book) was 16.5 years. It is important to note that our literature analysis includes review articles and similar publication types, rather than primary research studies. Accordingly, when a particular topic emerges in these analyses, it likely indicates that the issue had already achieved a certain degree of consolidation within the scientific discourse of the period.

Furthermore, we wanted to test the hypothesis that current neurobiology of mental disorders tends to “smallism,” defined as “the contention that mature cognitive science will explain in terms of the smallest aspects of physical reality” (Oliveira and Chemero, 2015, p. 14; Wilson, 2004). After an initial overview of the topical progression in the six editions of the *Principles*, we found a trend of an increasing interest in progressively smaller units: from brain areas to neurons to proteins to genes. We wanted to test whether this was the case in addiction. Overall, these two questions –the balance between various addiction hypotheses and the “smallism” issue– entail two trajectories in the discussion: one, so to speak, cross-sectional, where each edition of the *Principles* is compared with its corresponding state of the art; and one longitudinal, where the evolution of addiction can be discussed across the editions of the *Principles* (and, independently, in the state of the art of the different periods). This section first discusses the “cross-sectional” approach, then the “longitudinal.”

One overall conclusion that affects both approaches is that addiction was overlooked in Kandel’s manual until the fourth edition, published in 2000. However, it was mentioned as a significant societal problem since the beginning, so it may be inferred that neural science, according to the editors, did not have much to say about it until the end of the 20th century. However, the cross-sectional analysis reveals that the state of the art in the first three editions showed a high diversity of psychological and neurobiological hypotheses, already suggesting the role of catecholamines, the involvement of prosencephalic areas such as the medial forebrain bundle, and other reward-related structures. Furthermore, these hypotheses emphasized the importance of integrating biological, sociological, and psychological components, thereby embedding neurobiology within the context of genetic and epigenetic factors. Interestingly, the first edition already mentioned intracranial self-stimulation studies in the context of reinforcement and homeostasis, which were topics related to the neurobiology of addiction as early as the 1970s, but failed to make the connection in the manual. This could be due to the fact that combining homeostasis, reinforcement, and hedonic factors involved a thorough explanation of the anatomy and function of the hypothalamus, whose complexity may leave little room for novel topics, such as the link between homeostasis, reinforcement, and addiction. Before explicitly dealing with addiction in the fourth edition, the third edition includes very similar content to the previous ones. However, neurobiological research on addiction made substantial progress during the 1980s. As an example, Wise’s review in 1987 extensively demonstrates the involvement of the ventral tegmental area and nucleus accumbens in addiction (Wise and Bozarth, 1987), among other things, and this mental disorder is not mentioned in the *Principles* (1991, third edition) when the basal ganglia are explained. In conclusion, the lack of focus on addiction in the first three editions may be a consequence of describing reinforcement in connection with homeostasis and the hypothalamus, rather than making the move to link reward-related processes to the basal ganglia.

We do not have a definitive answer on why addiction was not included in the earlier editions. However, we hypothesize the following: In the first three editions, the chapter in which addiction was eventually incorporated was authored by Irving Kupfermann, a leading expert on feeding behavior, defense reflexes, motivation, and behavioral modulation in *Aplysia*. Given that the chapter addressed motivation and the limbic system, it is unsurprising that its primary emphasis was placed on mechanisms related to the maintenance of homeostasis. Concepts such as pleasure, reward, learning, and emotion were therefore framed mainly as processes supporting the regulation of normal behavior. In line with Kupfermann’s research background, which focused largely on fundamental behavioral mechanisms, pathological behavioral states such as addiction were not explicitly discussed. Moreover, his continued authorship of the chapter in the second and third editions suggests a strong conceptual continuity across these versions. In the fourth edition, however, authorship expanded to include the neuropsychopharmacologist Susan Iversen. This addition coincided with a broader shift in the chapter’s focus, with greater attention given to addictive processes. Although the editorial motivations behind this change cannot be established, Iversen’s expertise in the behavioral and neurobiological effects of psychostimulants, particularly amphetamines, may have contributed to a conceptual reorientation linking motivational and emotional systems with addictive states.

Indeed, the fourth edition, although briefly, discusses the role of dopaminergic pathways in reinforcement and addiction. This section is still included in a chapter about the hypothalamus, but the nigrostriatal pathway is highlighted as a crucial site for motivation. The bibliography is up to date, citing, for instance, Koob, Robbins, Everitt, Wise, or Schultz, including articles from the late 1990s. Therefore, its content is more aligned with the state of the art than previous editions. Even though addiction is not mentioned in the chapter on the basal ganglia, the role of the limbic loop (including the ventral striatum, ventral pallidum, ventral tegmental area, etc.) in motivated behavior is stated. Also, this edition goes deeper into psychological terms such as tolerance, craving, or dependence. Neurobiology is much more integrated into current hypotheses of addiction, even though the explanations are brief. The explanation of addiction definitely changed in 2012, when the fifth edition was published. A quick look at the index reveals ten different mentions of the topic (from animal models of addiction to relapses, plus five subtopics within “addictive drugs”). As explained above, drug abuse and addiction are presented as goal-directed behavior, which is at odds with most psychological hypotheses. However, in the text, it is stated that “The addicted person loses control over drug use –obtaining and using drugs come to dominate all other life goals” (Kandel et al., 2013, p. 1105). Neuroanatomically, it is focused on reward circuits and the role of dopamine in reward anticipation and consumption, after Wolfram Schultz’s experiments (Schultz, 1997). This section is still in the homeostasis and motivation chapter, although hypothalamic anatomy and function is mostly explained in previous chapters. Several diseases are mentioned in the basal ganglia chapter, although addiction is absent. However, chapter 66 presents the cellular mechanisms of implicit memory, and it is stated that “[the caudate nucleus] and the ventral striatum malfunction in a variety of diseases in which habit learning is disordered, including obsessive-compulsive disorder and addiction” (Kandel et al., 2013, p. 1482). This goes in line with Everitt and Robbins’ theory of addiction, who interpret it as a rigid and uncontrollable habit. Therefore, the fifth edition treats addiction extensively, although most of the descriptions were already present in the scientific literature for nearly two decades.

The sixth edition is marked by the independence of addiction from homeostasis. Chapter 43 distinguishes between goal-directed motivated behavior and drug addiction as a pathological reward state. Therefore, addiction is not presented as a goal-directed process anymore. For the first time, addiction is also explained in the basal ganglia chapter as a dysregulation of motivational selections where there is an exaggerated salience of certain stimuli. Strikingly, in times where behavioral addictions seem to be as pervasive as substance abuse, the “addiction” entry in the index is substituted by “Drug addiction” and “Drugs of abuse.” However, a final section in chapter 43 deals with “natural addictions,” although this is just outlined as a promising field of research. Compared to the current state of the art, there exists the risk of overlooking current psychological and neurobiological hypotheses of behavioral addictions, as it happened in the first editions with addiction in general. Thus, we recommend a more in-depth analysis of these conditions, as some systematic reviews and meta-analyses indicate that their prevalence exceeds 10% (Alimoradi et al., 2022). Finally, the comparison between the *Principles* and the state of the art reveals an interesting mismatch in the interpretation of the Olds and Milner experiment about intracranial stimulation in rats (Olds and Milner, 1954). The latter editions of the manual keep presenting it as a repeated self-stimulation because it feels pleasurable to the animal. However, scientific evidence since the 1990s suggest that animals self-stimulate because they feel the urge to do it (they “want” it), not because they experience pleasure (they do not “like” it) (see Kringelbach and Berridge, 2012 for a summary of this change of perspective).

Concerning the “longitudinal” analysis of addiction research, the evolution in the *Principles* can be summarized in the following points: (1) recognizing addiction as a neurobiological (and not just a societal) issue; (2) linking the effect of some drugs (i.e., cocaine) to dopamine and reinforcement; (3) disentangling addiction from homeostasis and hypothalamic function, bringing it closer to mesolimbic circuits and learning; (4) increasing fine-grained descriptions of the molecular processes involved in addiction; (5) suggesting, as a future field of research, that the neural bases of substance addiction may be the same as behavioral addictions. In the scientific literature preceding each edition, the psychological hypotheses of the 1940s and 1950s were progressively applied to neurobiological findings. In the early 1980s, dopamine was introduced as the most plausible candidate to explain addiction. These hypotheses were also ecological, admitting that there should be genetic, neurochemical, and social factors that made some individuals more vulnerable to addiction. Another evolving topic across time is the dual perspective on addiction. All hypotheses employ a dialectic approach to provide a broader perspective on the issue: the shift from positive to negative reinforcement over time, the transition from liking to wanting, from goal-directed behavior to compulsive habits, and so on. Interestingly, whereas descriptions in the *Principles* progress toward more detailed molecular processes, as if more detailed meant more precise, current scientific approaches tend to integrate genetic, epigenetic, neurobiological, and social factors, just as they did decades ago. In other words, molecular descriptions do not come at the cost of missing the bigger picture: they are used to enrich it. In our opinion, the handbook should follow the same track and incorporate the latest advances in neurobiology within a holistic framework where addiction is viewed as a biological, psychological, and social mental condition.

Even though this is an extensive study of the history of addiction neurobiology in the last half century, our work is limited to Kandel’s handbook and scientific reviews in the literature. Another possible strategy would be to compare the *Principles* with other textbooks focused on addiction, such as those within the scope of pharmacology, for example (like Goodman and Gilman’s handbook, Brunton and Knollmann, 2023). To have a more restricted field, we decided to analyze just one handbook and compare it with the state of the art. We encourage future research to compare the neurobiology of addiction between different textbooks. Also, it should be taken into account that our historical review is not intended to show the visibility or impact (Petrovich and Viola, 2024) of the state-of-the-art literature on each edition of Kandel’s textbook. We do independent analyses for both sources, and compare their contents generally. For future research, it would be interesting to study how the current state-of-the-art will eventually be integrated into the possible next edition of Kandel’s handbook.

In conclusion, we hope that this historical and critical review helps the reader gain a deeper understanding of the neurobiology of addiction and inspires future research. We would like to close with the final paragraph of the Preface included in the 6th edition of the *Principles*, as a tribute and acknowledgment to Eric Kandel and his collaborators, and to express our main goal with this article:

In writing this latest edition, it is our hope and goal that readers will emerge with an appreciation of the achievements of modern neuroscience and the challenges facing future generations of neuroscientists. By emphasizing how neuroscientists in the past have devised experimental approaches to resolve fundamental questions and controversies in the field, we hope that this textbook will also encourage readers to think critically and not shy away from questioning received wisdom, for every hard-won truth likely will lead to new and perhaps more profound questions in brain science. Thus, it is our hope that this sixth edition of *Principles of Neural Science* will provide the foundation and motivation for the next generation of neuroscientists to formulate and investigate these questions (Kandel et al., 2021, p. xlii).
